# Prejudice Against Immigrants Symptomizes a Larger Syndrome, Is Strongly Diminished by Socioeconomic Development, and the UK Is Not an Outlier: Insights From the WVS, EVS, and EQLS Surveys

**DOI:** 10.3389/fsoc.2019.00012

**Published:** 2019-02-26

**Authors:** M. D. R. Evans, Jonathan Kelley

**Affiliations:** ^1^Department of Sociology and Applied Statistics Program, Nevada Agricultural Experiment Station, University of Nevada, Reno, NV, United States; ^2^Department of Sociology, University of Nevada, Reno, NV, United States; ^3^International Survey Center, Point Marka, NSW, Australia

**Keywords:** prejudice, social distance, public opinion on immigrants, socioeconomic development, Brexit, UK, trends

## Abstract

Public attitudes toward immigrants in the UK, especially prejudice against them, form a strong theme in retrospective media postmortems emphasizing the uniqueness of Brexit, yet similarly hostile public opinion on immigrants forms a recurrent theme in populist politics in many European Union nations. Indeed, if UK residents are not uniquely hostile, then the UK's exit from the EU may be only the first symptom of proliferating conflicts over immigration that will plague EU nations in future years. A well-established symptom (or consequence) of prejudice—aversion to outgroups as a neighbors—shows that prejudice against immigrants, other races, Muslims, Hindus, Jews, and Gypsies are all relatively low in the UK. This is as expected from the general decline of prejudice and social distance with socioeconomic development, demonstrated here in broad perspective across many countries. Indeed, UK residents are about as prejudiced against each of these ethno-religious outgroups as are their peers in other advanced EU and English-speaking nations, and much less prejudiced than their peers in less prosperous countries. Confirmatory factor analysis supports the view that a single latent ethno-religious prejudice generates all these specific prejudices, so it is not specific experiences with any one of these groups, nor their specific attributes, that are the wellspring of this deep-seated underlying prejudice. Replication using other measures of prejudice and another cross-national dataset confirms these findings. Data are from the pooled World and European Values Surveys (over 450,000 individuals, 300 surveys, and 100 nations for this analysis) and from the well-known European Quality of Life surveys. Analysis is by descriptive, multilevel (random intercept, fixed effects), and structural equation methods.

## Introduction

Do values and attitudes about immigration and ethnic and religious diversity set the United Kingdom apart from the European Union? Clearly, the prospects for constructive engagement between nations negotiating a common labor market are better if these attitudes are shared, especially given EU rules about open migration. Prejudice complicates the employment process (Neckerman and Kirschenman, 1991; Heath et al., 2008; Kelley and Evans, 2015) as well as the political process (Breznau and Eger, 2016; Wagner and Meyer, 2016). A crucial issue for a “common labor market” is a civil residential environment for people working away from their home country, so it is especially important to know how the natives of a country feel about immigrant workers as neighbors. Indeed, how one feels about having members of potential outgroups as neighbors is a classic social distance measure with a distinguished intellectual pedigree (Bogardus, 1933). There is a wide range of perfectly legitimate interpretations of the relationship between the concepts of “prejudice” and “social distance,” ranging from seeing “social distance” as a symptom or indicator of prejudice to seeing normatively endorsed social distance as a cause of prejudice, to seeing prejudice as a cause of social distance. We will here adopt the Park approach (Park and Burgess, 1921; Park, 1924) that social distance is a symptom or indicator of prejudice, in part because the research seeking to establish a causal direction remains inconclusive.

But if attitudes about immigration, ethnic and religious diversity do not set the UK apart—if Britain is not unique within the EU—then Brexit may not reflect circumstances unique to Britain. It might instead be that Brexit is only the first symptom of wider difficulties that will come to plague EU nations in future years. Of course, there are many other ways in which Britain is known to be “exceptional” in the European context (Castles, 2010; Evans and Kelley, 2017, 2018), so a similarity between Britain and “the Continent” on one dimension, such as aversion to “outgroups” different in nationality, ethnicity, or race does not necessarily imply similarities in other domains of culture.

Prior research has long shown that some degree of anti-immigrant prejudice is present in all European countries, varying widely among them (Scheepers et al., 2002; McLaren, 2003; Zick et al., 2008a; Davidov and Semyonov, 2017). We extend that research to the overseas Anglophone countries and, beyond them, to the world at large.

Thus, this paper explores ethno-religious prejudice in comparative, cross-national perspective, with special reference to the UK. We compare the UK to the European Union (Scheepers et al., 2002; Zick et al., 2008a; Gorodzeisky and Semyonov, 2015), to the UK's culture group—the other Anglophone countries (Kelley and Evans, 1995; Inglehart, 2008; Inglehart and Welzel, 2010), and to the rest of the world. For clarity, we will take into account the effect of socioeconomic development, as indexed by GDP per capita, on various aspects of prejudice (Blalock, 1967; Inglehart, 1981, 1990, 1997; Ruist, 2016). Within the EU, we distinguish the post-Communist countries from others, as their patterns of prejudice may differ (Kunovich, 2004). This does not imply that these are the only potential influences of social context on prejudice(s), but rather takes the Maslowian perspective (Maslow, 1943) that socioeconomic development is at least one root cause of many attitude and value trends.

Recent research on Europe finds moderately strong links across prejudices against different targets in the general domain of ethnicity and religion—immigrants, people of different race, ethnicity, religion, or nationality (Zick et al., 2008a; Gorodzeisky and Semyonov, 2015). This raises the question of the degree to which ethno-religious prejudices are a patchwork of unrelated attitudes and to what degree they reflect an underlying schema, a single approach or orientation that generates the apparently specific attitudes (Lemmer and Wagner, 2015). The “cognitive turn” in cultural sociology suggests that each of these apparently distinct prejudices is a kind of symptom or indicator of a single underlying schema of ethno-religious prejudice (DiMaggio, 1997; Brubaker et al., 2004). According to this line of reasoning, culture is neither a coherent whole with a unitary logic nor a happenstantial midden of unrelated attitudes. Instead it is at least a stew with coherent integrated chunks (in our example, schemas) that may or may not be integrated with each other into a casserole. Supportive empirical evidence for this approach has been reported for Germany (Zick et al., 2008b).

Prior theory and research raise three possibilities: (1) Attitudes toward immigrants and toward different ethnic and religious groups are each a separate matter deriving from specific experiences of contact and of local feeling; (2) These attitudes form a coherent whole: There is an underlying latent variable of prejudice toward immigrant, ethnic, and religious outgroups that is distinct from prejudice toward other outgroups or other disempowered groups, e.g., LBGTQ, disabled, etc. (DiMaggio, 1997; Guimond et al., 2003; Brubaker et al., 2004; Lemmer and Wagner, 2015); and (3) These attitudes form a coherent whole that covers negative sentiment toward all outgroups and disempowered groups, perhaps reflecting prejudice as a generalized personality trait (Allport, 1979 [1954]; Stangor et al., 1991; Bergh et al., 2016). Our study was largely an exploratory, inductive one, endeavoring to examine whether Britain was distinct on a wide variety of ethno-religious prejudices, to discover whether these prejudices hang together worldwide, and to assess the impact of socioeconomic development on ethno-religious prejudice.

We also include an exploration of changes over time, net of our substantively measured variables. Of course, time is not itself a social force. Rather, it represents the influence of countless unmeasured social forces, so our responsibility as social scientists is to specify the relevant substantive influences that lurk inside the label “time” or “changes over time.” Nonetheless, exploring changes over time may provide clues about which substantive influences are at work. Given our inductive approach, formal hypothesis development would be *post-hoc* and hence inappropriate for an introduction. We do develop some working hypotheses for future deductively-oriented research in the section Discussion.

### A Note on Terminology

This article focuses on negative feelings toward immigrant, ethnic, and religious minority groups, which we shall call “prejudice,” but we recognize that there are nearly as many specific uses of the word, and of associated terms such as “social distance” and “aversion,” as there are scholars who use them. “Group-focused enmity” has also been proposed (Zick et al., 2008b), but implies a very strong magnitude and we need a term that can encompass sentiments ranging from very mild to very intense. We will use “prejudice” in its broad sense to mean negative feelings, negative sentiment, aversive emotions, etc. and shall consider “social distance” to be a symptom or indicator of prejudice (see also, e.g., Storm et al., 2017). “Social distance,” too, ever since its invention (Park and Burgess, 1921; Park, 1924) plays many different roles in the sociology, anthropology, and psychology of minority-majority groups relations, ranging from a strictly institutional one (degree of normatively and/or legally allowed contacts between social groups) to a strongly affect-based one (desire for lack of contact, degree of desired separation). Social distance as institutionally defined could be a cause of prejudice via system justification mechanisms; social distance as emotion could be either an indicator of prejudice or a consequence of prejudice. The indicator we will explore, desire to avoid having members of various ethnic and religious outgroups as neighbors, is familiar from its use as a component item in the well-known Bogardus social distance scale (Bogardus, 1933). As one social history of the matter put it, “The scale was developed by Emory Bogardus in 1924 and is still widely used in measuring prejudice” (Wark and Galliher, 2007).

## Data, Measurement, And Method

### Survey Data: WVS, EVS, EQLS

Data are from the World Value Study and European Values Study datasets (EVS, 2015; WVS, 2015) pooled for all available years (Díez-Medrano, 2011). This is a splendid and highly regarded dataset, well-documented on the two organizations' websites.

In the full dataset there are over 340 surveys, over 100 countries, and over 500,000 individual respondents. The several questions analyzed here were asked in varying numbers of surveys with therefore varying numbers of respondents (described in the text). For example, our key variable, prejudice against immigrant workers, was asked in 327 surveys from 101 countries, with 448,269 individual respondents. We treat all countries as equally weighted units (e.g., Hungary and United States both have weights of 1) in the multilevel analysis, in the scatterplots and the estimation of the fit lines connecting prejudice with socioeconomic development. Ockham's Razor dictates that the simplest method is to be preferred unless additional complexity demonstrably reveals important evidence that would be overlooked with the simpler method, so, like much other recent research using these and similar datasets, we do not re-weight the individual-level cases (Esping-Andersen and Nedoluzhko, 2017; Kelley and Evans, 2017; Breznau and Hommerich, 2018; Evans and Kelley, 2018; Fernandez and Jaime-Castillo, 2018; Ignacz, 2018; Miranda et al., 2018; Ng and Diener, 2018; Roex et al., 2018).

For this analysis, we dropped all nations with <1 million citizens, several city-states (Hong Kong, Luxemburg, Singapore) on the grounds that prejudice-generating processes could be different in such relatively intimate settings, and one nation that did not ask the relevant questions (Israel).

We also provide an auxiliary analysis replicating the key result on an alternative dataset, the well-known European Quality of Life Survey of 2011–2012 with representative nationwide surveys in 29 countries and *N* = 66,795 individuals on the question of interest. It is well-documented on-line (www.eurofound.europa.eu).

For some variables, Northern Ireland has a separate dataset, and for others it is pooled with Great Britain. For simplicity, the datasets containing Great Britain will all be labeled “UK” in the graphic displays in the paper (represented by a green dot); technically most of them are UK, but some are GB. In all cases where they are GB, Northern Ireland is shown separately as a blue dot (like the other Anglophone countries).

### Measurement

The key questions on prejudice are about objecting to a member of a possible outgroup as a neighbor. This is an element of the classic Bogardus social distance scale (Bogardus, 1933). Also, recent theorizing about ethnicity-as-cognition would posit that the stimulus of being asked about different groups as neighbors elicits mental “scripts” in which the survey respondent calls to mind likely sequences of events involving a neighbor belonging to the group in question (Brubaker et al., 2004). For our purposes, the key item is “immigrant/ foreign workers” (v37), and items we also examine include “people of a different race” (v35), and “people of a different religion” (v39) as well as Gypsies, Hindus, Jews, and Muslims (from “add on” versions or other years). Of course, immigrant or foreign workers theoretically need not be ethnically or religiously distinct, but, in practice, most have a different native language, which the general population seems to regard as a marker of, or equivalent to, ethnicity.

The verbatim from the World Value Study Wave 5 is (WVS, 2014):

**Table:** 

*(Show C ard D)*			
On this list are various groups of people. Could you please mention any that you would not like to have as neighbors? *(Code au answer for each group)*:			
		**Mentioned**	**Not mentioned**
V34.	Drug addicts	1	2
V35.	People of a different race	1	2
V36.	People who have AIDS	1	2
V37.	Immigrants/foreign workers	1	2
V38.	Homosexuals	1	2
V39.	People of a different religion	1	2
V40.	Heavy drinkers	1	2
V41.	Unmarried couples living together	1	2
V42.	People who speak a different language	1	2
V43.	*(Optional: minority relevant to given country, write in): ______*	1	2

Unfortunately, all these items are dichotomies so, as has long been known, measurement is crude and random measurement error is greater than if they had been measured on a 5-category or 7-category scale (Gjeddebaek, 1968; Heitjan, 1989; Haitovsky, 2001). Nonetheless, the “neighbor” concept is a strong one, so there are good prospects for learning something from these items despite the crude measurement.

Somewhat different lists of groups were offered in different surveys, varying both by nation and by date of survey, but “Immigrants/foreign workers” and “People of a different race” were almost always included. The standard European Values Study wording is very similar: “On this list are various groups of people. Could you please sort out any that you would not like to have as neighbors.” The two wordings yield closely similar results.

Despite valiant attempts at comparability, there is one major error. The French 2006 WVS used a variant wording that produces a huge spike in mentioning “immigrants/foreign workers” and the other groups that could be compared (for details see http://www.worldvaluessurvey.org/WVSDocumentationWV5.jsp). The variant wording looks innocuous (available on the WVS website) but the strongly out-of-line results compared to other French surveys both before and after mean that it cannot be used as equivalent to the standard question, so we have omitted the French 2006 WVS.

The Hungarian survey asked this as a series of separate questions (which of course produces higher reliability data), rather than in the standard format, but this does not appear to introduce any distortions: Correlations among items and with other variables are within a plausible range, based on the other countries and the proportions/means are reasonable, so we have retained the Hungarian data.

Other groups are included in some waves and some countries (details in Appendix A). Specifically of interest to us are Gypsies, Hindus, Jews, and Muslims.

We will also use some individual-level predictors in the structural equation model described below. One of the longest established of these effects is education enhancing tolerance (Stouffer, 1955), we will measure it in full-time equivalent years of education completed. In some instances, this must be estimated from the highest level of education completed. Age, gender, and (relative) income have ambiguous effects in prior research, but they are never large (Quillian, 1995; Semyonov et al., 2006; Rustenbach, 2010; Gorodzeisky and Semyonov, 2015). Religion's effects have been controversial since the beginning (Allport and Kramer, 1946; Lenski, 1961; Scheepers and Eisinga, 2015), but our purpose is not to evaluate the competing theories about it, but rather simply to use religiosity, as indexed by a 4-item religious belief scale (Kelley and de Graaf, 1997), as a criterion variable.

### Methods: Visualization, OLS, and Multilevel Analysis

We explore multiple specific examples of ethno-religious prejudice, considering the proportion who would reject specific groups as neighbors by country to provide a kind of social epidemiology of prejudice comparing the UK to peer nations in the EU, to the overseas Anglophone nations (the UK's sometimes obstreperous offspring which inherited traditional English law and institutional arrangements), to poorer EU nations, and to a broad representation of other nations around the world. For the EU, we also provide a regression line from a simple aggregate model predicting the proportion shunning specific groups as neighbors from the level of socioeconomic development of the nation (allowing both a linear term and a quadratic term).

We then assess how close the UK is to the level of prejudice (against a particular group) that is typical of EU countries at approximately the same level of development. The robustness of aggregate analyses of this kind is sometimes influenced by seemingly minor decisions about missing data, variable definition, and functional form (Breznau, 2016), so we are fortunate to have the large number of surveys in the WVS/EVS family, over 300, available for this project. This provides a kind of social epidemiology of prejudice (Sperber, 1985), a rich context in which to consider the prejudice of central interest to this paper.

Having set the context, we then consider the level of prejudice against immigrant/foreign workers, our main focus, using this same approach.

We next turn to the question of the degree to which ethno-religious prejudices are a patchwork of unrelated attitudes and to what degree they are reflect an underlying schema, a single approach or orientation that generates the apparently specific attitudes. Confirmatory factor analysis of the prejudice items as a latent dependent variable in a structural equation model is an appropriate statistical method (Bollen, 1989; Treiman, 2009). Because we have used multiple imputation of missing data, fit indices are not appropriate, but the inter-item correlations, factor loadings and correlations with criterion variables all support the view that all the items tapping negative sentiment toward foreigner, ethnic, and religious groups all tap one underlying dimension, one latent variable, and that this latent variable is distinct from negative sentiment toward other outgroups.

As well as having strong associations with each other, the observed items measuring a latent variable/construct must have closely similar associations with criterion variables—variables not in the scale but which might reasonably be expected to be among its causes or consequences. As noted above in the Measurement section, the criterion variables we use are age, gender, education, income, and religiosity.

Following current best practice (Kelley et al., 2017), we do not group-mean-center the variables in our multilevel analyses.

We provide several structural-equation, OLS, and multilevel analyses depicting the impact of GDP (allowing curves) and assessing whether the UK is different net of GDP and individual characteristics. For some of these we provide graphs of predicted values of the means using whole-population standardizations that show the predicted means on the response variable (prejudice) across the range of the predictor variable of interest whilst holding the other predictor variables constant (based on the model described in conjunction with the graph). We use OLS for the country-specific models of change over time (since there was no pattern evident in the pooled file). The detailed equations are in Appendix B.

In addition to our main analysis, we provide two sensitivity analyses applying our model to two response variables with different wording but in the same conceptual domain and an additional sensitivity test to see whether the GDP effect changes when % foreign in the country is taken into account.

#### Causality

GDP per capita at parity purchasing power not only expresses socioeconomic development differences among countries, but it also evolves within countries. Several other of the predictor variable we will use have also show major shifts over the period under consideration: education has risen, populations have aged, the sex composition of societies has shifted toward women, but religious belief holds steady or shifts erratically. Our estimates of the effects of these variables are unbiased unless it can be shown that they proxy for omitted variables. We know that relative income, education, and GDP are all connected with actual individual income (for example, in dollars at parity purchasing power) which is unmeasured in these surveys. But education is clearly causally prior to income (both measured at the individual-level), so its effect is unbiased, provided that we interpret it as a total effect potentially including an indirect effect through income rather than a direct effect.

## Results

### Results, Part 1: The Context: Ethno-Religious Prejudices in Detail, as a General Syndrome, and How Attitudes Toward Immigrants Fit In

We begin by setting the context, inquiring about various aspects of ethno-religious prejudice to get the big picture before going on to prejudice specifically against migrants/foreign workers. Details are in Appendix A.

#### Prejudice Against People of a Different Religion

**Figure 1:**
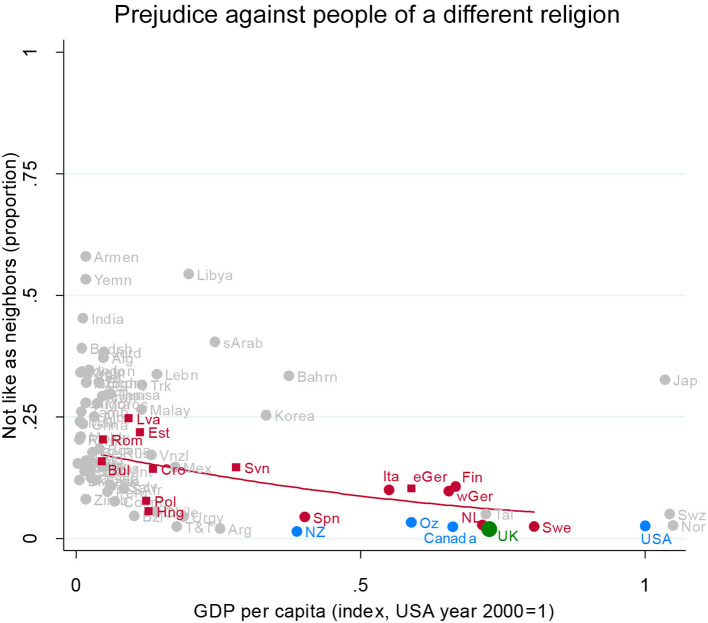
Prejudice against people of a different religion. Attitude in the UK (green), in other English-speaking nations (blue), in EU nations (red squares for ex-Communist, circles for others), and other nations (gray). Fit line just for the EU, excluding the UK. GDP per capita (index, USA year 2000 = 1). Source: World Value/European Value Studies 1981–2014 (193, 644 individuals for this analysis).

We start with prejudice against people of a different religion. For each country, we calculated the percent saying that they would not like to have people of a different religion as neighbors (Figure 1, below). The UK is shown as a green dot; the other Anglophone countries are shown in blue; EU countries other than the UK are in red; and other countries are in gray. Further details are in Appendix A.

To clarify the relationship, we have arrayed the countries from left to right according to their socioeconomic development as indexed by their GDP per capita—a major influence on prejudice and one in which there is substantial variation in the EU. The red line shows the statistical relationship between GDP per capita and prejudice against people of a different religion for the EU (leaving aside the UK).

Of key interest here is whether the UK is like other advanced countries in the EU, or whether it is like its cultural kin, the other Anglophone countries, or whether it is something quite distinct.

Prejudice against people of a different religion is very low in the UK (green dot, partially obscured between Australia and Sweden): under 5% would dislike having a neighbor of a different religion. In this, the UK is closely similar to the other Anglophone countries (blue dots).

Prejudice against a neighbor of a different religion is very diverse in the EU (red dots). At the same socioeconomic level as the UK, Sweden is just as unprejudiced as the UK; Spain, Italy, Finland, and Germany are a little more prejudiced.

Looking across the graph from left to right shows that there tends to be more diversity in prejudice among the developing nations and less among the advanced nations (although Japan is a prejudiced outlier, as is well-known). The scatterplot narrows as GDP per capita rises. We also see a decline in prejudice across levels of GDP within the EU: The downward sloping red line shows prejudice declining from around 20% who would shun neighbors of a different religion in the poorest EU countries (such as Bulgaria and Latvia) to around 10% in Germany, Italy, and Finland. Poland and Hungary are exceptions, showing the low levels of prejudice typical of much richer EU nations.

All in all, if in fact prejudice against people cleaving to a different religion matters to labor mobility in the EU and to local resistance to a “common market” workforce, then the UK—and indeed all the advanced countries in the EU—hold a common, low-prejudice outlook. Details on other nations (mostly unlabeled dots in Figure 1) are in Appendix A.

This warrants a closer look because it is possible that people might feel that “other religions” in general are acceptable, but they might still feel prejudiced against specific religions. In addition, many Muslim immigrants are visually identifiable in Europe and the overseas Anglophone countries, so there could be an ethnic component here as well. How do members of the general population feel about the possibility of having a Muslim neighbor? (Note that we omitted Turkey from the analysis of prejudice against Muslim neighbors, since our focus is on minority groups).

#### Prejudice Against Muslims

**Figure 2:**
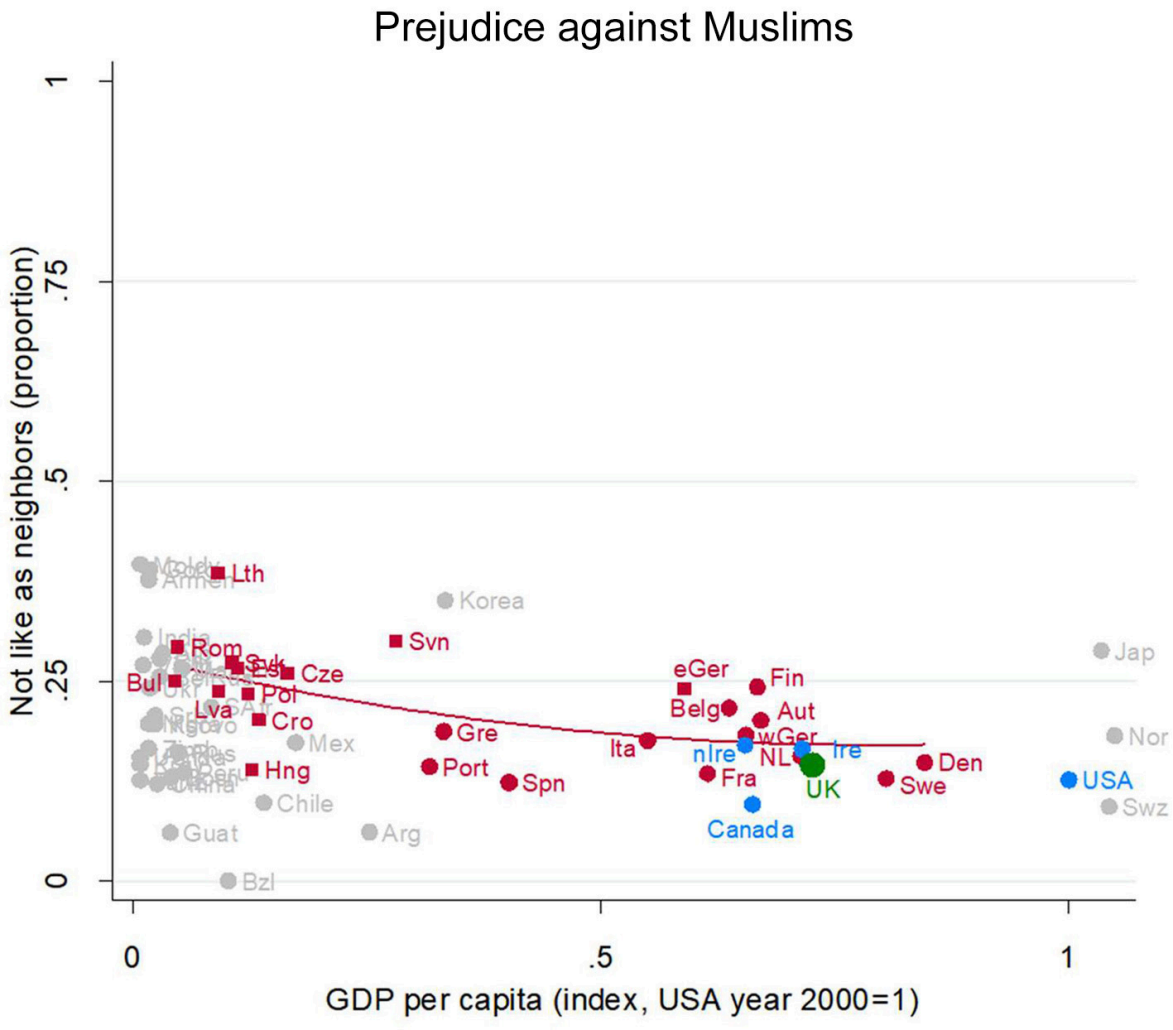
Prejudice against Muslims. Attitude in the UK (green), in other English-speaking nations (blue), in EU nations (red squares for ex-Communist, circles for others), and other nations (gray). Fit line just for the EU, excluding the UK. GDP per capita (Index, USA year 2000 = 1). Source: World Value/European Value Studies 1981–2014 (196, 569 individuals for this analysis).

The level of prejudice against Muslims in the UK (Figure 2, green dot, partially obscured, near Sweden and Canada) is very similar to other countries at the same level of development [see also (Bulmer and Solomos, 2010)]—indeed its very close proximity to the regression line shows that UK opinion is strongly typical of equally rich EU countries such as Italy, Germany, Austria, and the Netherlands, with Sweden perhaps a fraction less prejudiced but Finland and East Germany slightly more. Moreover, on this aspect of prejudice, the UK is also very similar to the other Anglophone countries.

Here again we see a development gradient, with prejudice somewhat higher among the poorer EU countries and lower among the richer EU nations. Looking across the whole array of nations also suggests some degree of convergence accompanying socioeconomic development.

Thus, the UK is very ordinary for its level of development in having a relatively low level of prejudice against Muslims. It is similar to comparable EU nations (including Germany) and similar to the other Anglophone countries. Again, further details are in Appendix A.

#### Prejudice Against Hindus

Hinduism is another “foreign” religion in Europe and the overseas Anglophone countries, and many of its adherents are visually distinctive. But unlike for Muslims, it does not have (at least in these countries) an association with terrorism. So, it is interesting to compare to the foregoing views about Muslims.

**Figure 3:**
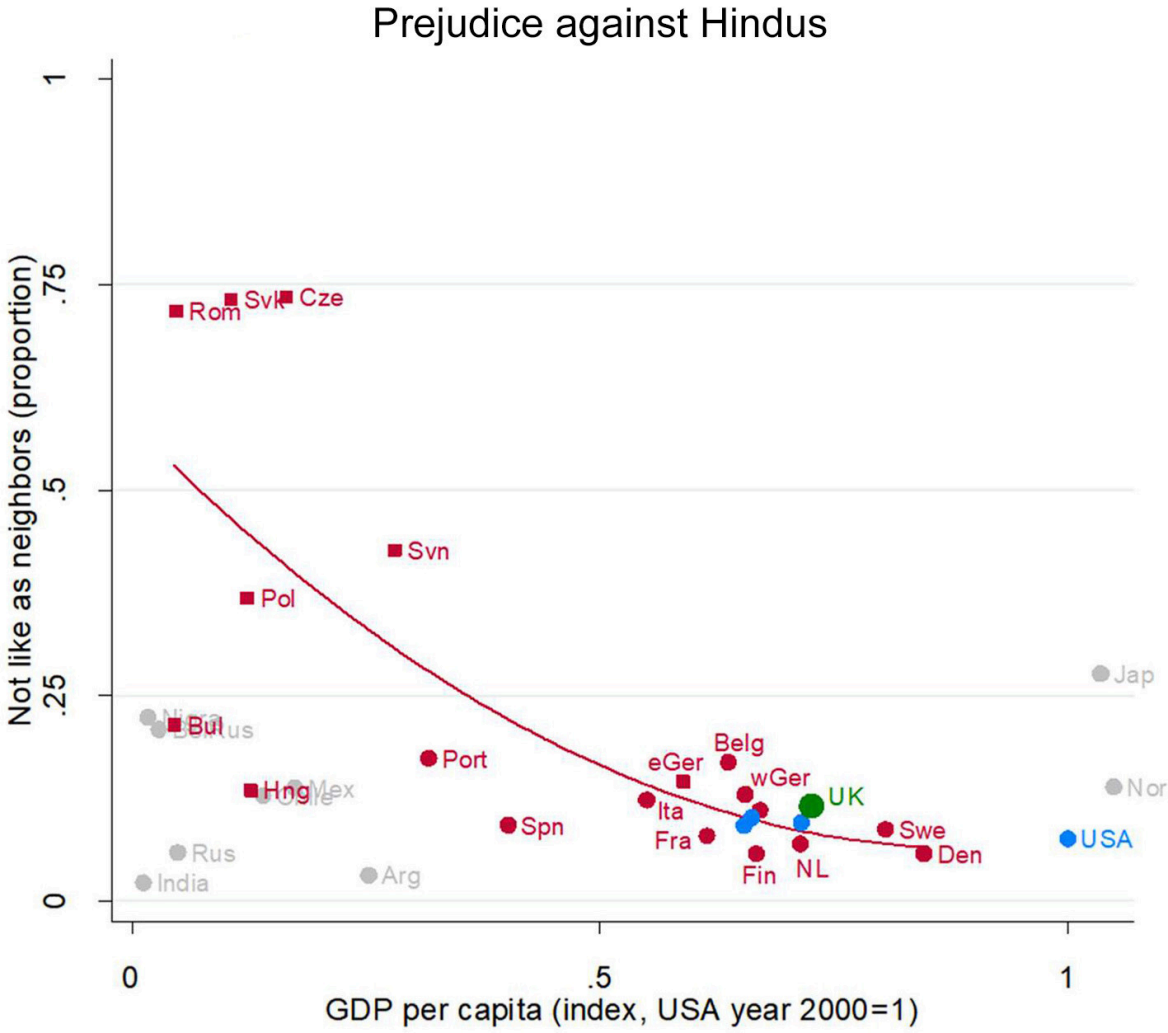
Prejudice against Hindus. Attitude in the UK (green), in other English-speaking nations (blue), in EU nations (red squares for ex-Communist, circles for others), and other nations (gray). Fit line just for the EU, excluding the UK. GDP per capita (index, USA year 2000 = 1). Source: World Value/European Value Studies 1981–2014 (49, 594 individuals for this analysis).

Here again, the UK is right where we would expect for an EU country at its level of development: The UK's green dot (partially obscured, between Belgium and Sweden) sits very near the regression line (Figure 3). Shunning a Hindu neighbor is very rare in all the rich EU countries. So too in the Anglophone countries (blue dots).

Here, there is a very steep development gradient in the EU (red line), with Romania, Slovakia and the Czech Republic being hugely more prejudiced against Hindus than other poor to middling EU countries. (Fewer countries asked this question, so there are fewer gray dots which makes it difficult to say what is going on outside the EU).

#### Prejudice Against Jews

**Figure 4:**
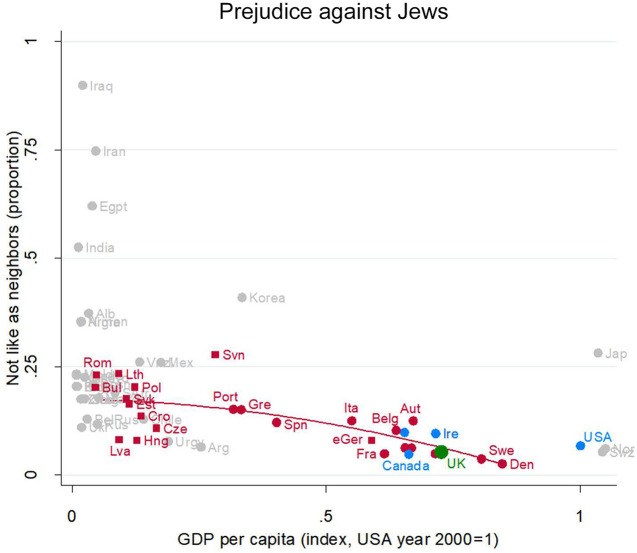
Prejudice against Jews. Attitude in the UK (green), in other English-speaking nations (blue), in EU nations (red squares for ex-Communist, circles for others), and other nations (gray). Fit line just for the EU, excluding the UK. Source: World Value/European Value Studies 1981–2014 (186, 218 individuals for this analysis).

Jews have long been the victims of prejudice in Europe, but in recent decades more than 90% in all the rich European countries would not object to a Jewish neighbor (Figure 4). In this, the UK (green dot, partially obscured just below Ireland) is again exactly where we would expect an EU nation at its level of development to be. People in the EU are much less prejudiced against Jews as neighbors than they are against Muslims, and this holds across all levels of development in the EU.

Prejudice against Jews is very low in all Anglophone nations (blue dots) but is varied and occasionally very high in poor non-EU nations.

#### Racial Prejudice

**Figure 5:**
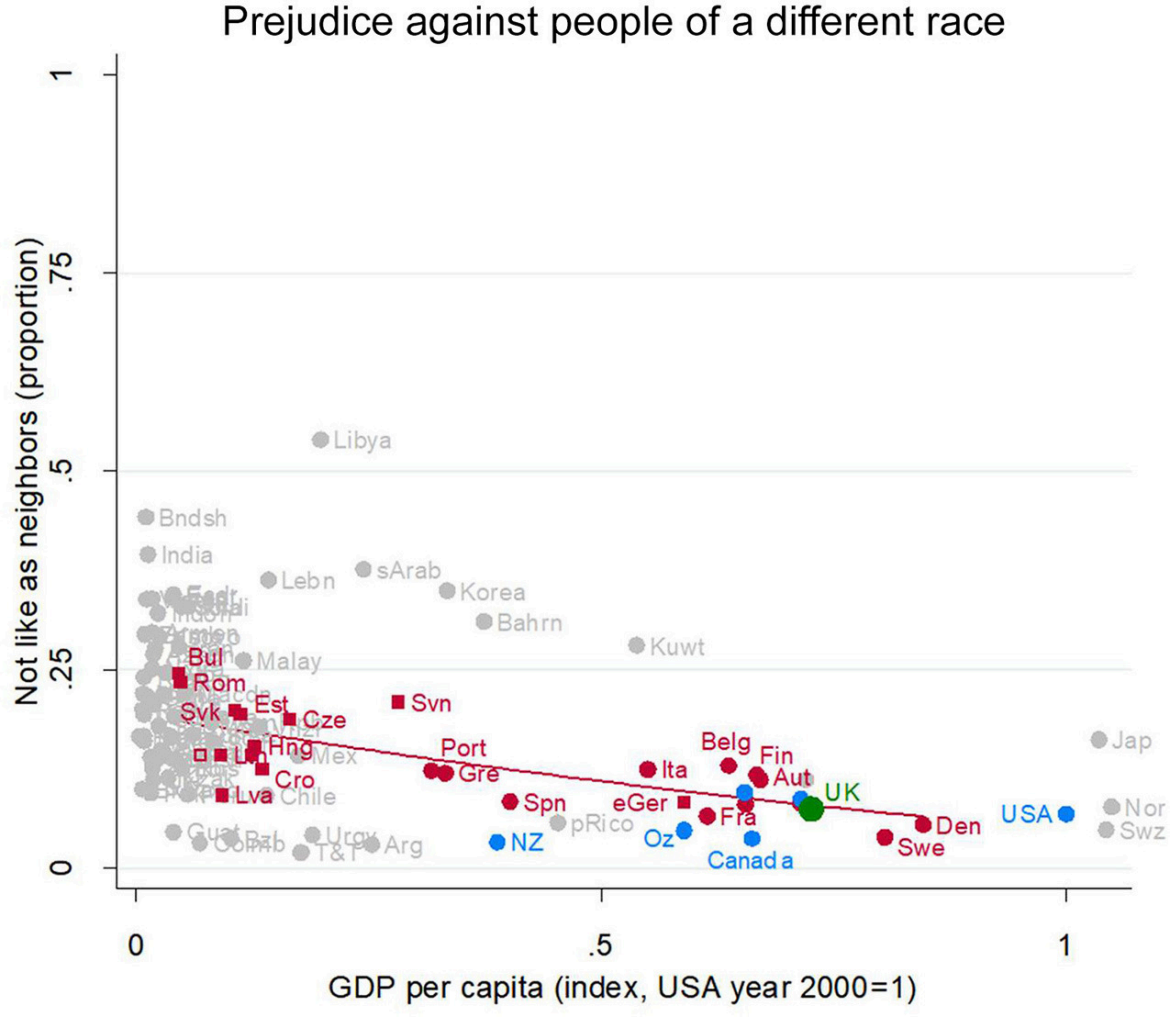
Prejudice against people of a different race. Attitude in the UK (green), in other English-speaking nations (blue), in EU nations (red squares for ex-Communist, circles for others), and other nations (gray). Fit line just for the EU, excluding the UK. GDP per capita (index, USA year 2000 = 1). Source: World Value/European Value Studies 1981–2014 (451, 824 individuals for this analysis).

Prejudice is fairly similar against people “of a different race.” It declines from around 20% at the less developed end of the EU to under 10% in the UK, Germany, Austria, Sweden, Belgium, the Netherlands and peer countries (Figure 5).

Here again, the UK has the low levels of prejudice typical of an EU country at its level of socioeconomic development: Its green dot (partially obscured, just above Canada) is right on the red regression line representing the relationship between socioeconomic development and prejudice for EU countries. This is also very close to the level of prejudice in the US and other Anglophone countries.

#### Prejudice Against Gypsies

**Figure 6:**
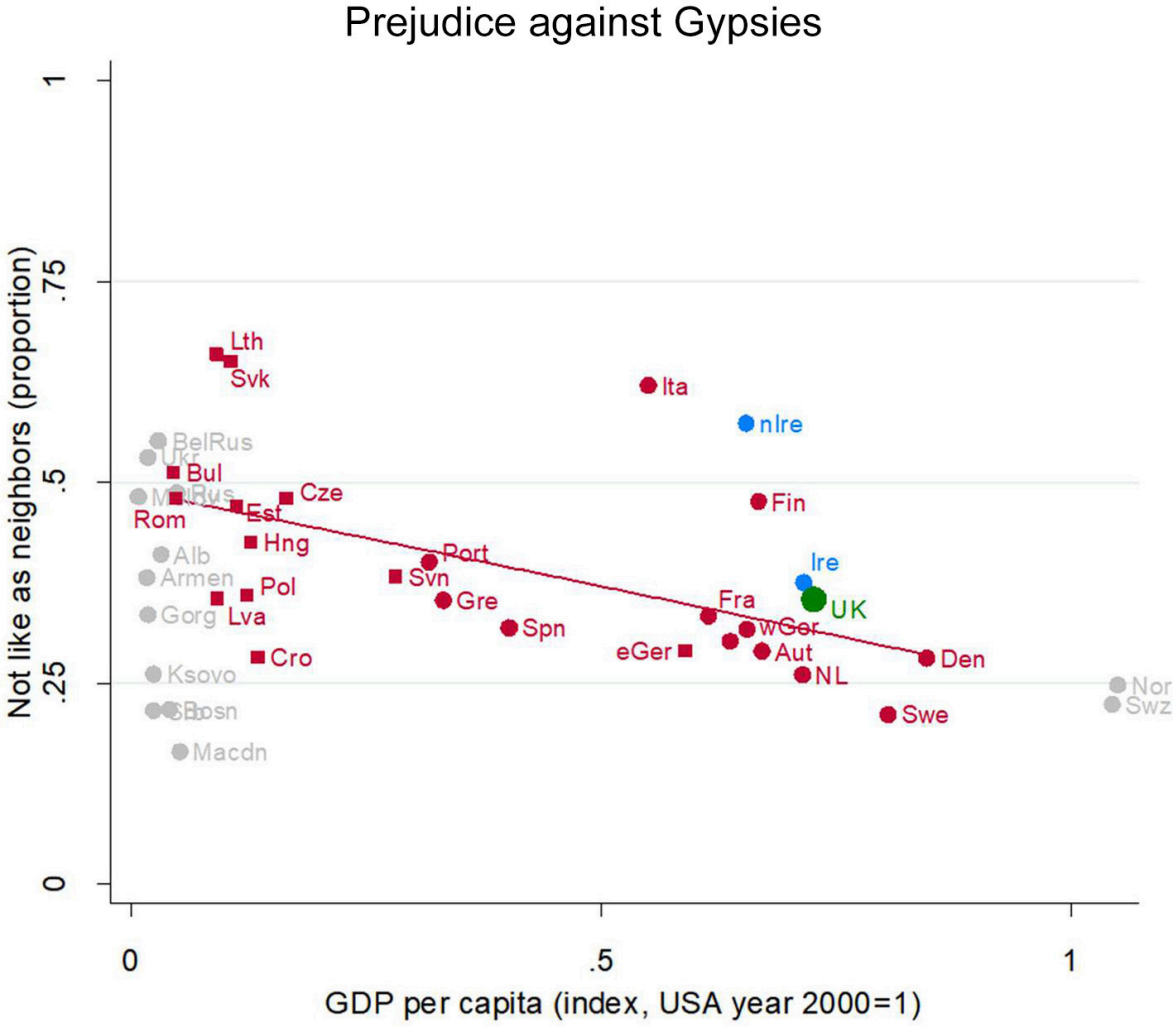
Prejudice against Gypsies. Attitude in the UK (green), in other English-speaking nations (blue), in EU nations (red squares for ex-Communist, circles for others), and other nations (gray). Fit line just for the EU, excluding the UK. GDP per capita (index, USA year 2000 = 1). Source: World Value/European Value Studies 1981–2014 (105, 258 individuals for this analysis).

But at least one ethnic group faces more prejudice: Gypsies. Nearly 40% of UK people would object to a Gypsy neighbor (Figure 6, green dot, partially obscured, near Ireland). That is nearly twice as many as would object to a Muslim neighbor. In this, they are again similar to their EU peers at the same level of development—a bit higher than the Austrians and the Dutch, a bit lower than the Finns.

There is a strong development gradient, from prejudice levels around 50% among the poorest EU nations dropping to around 25% in the richest. This question was only asked in Europe (including Russia), so there are fewer possible comparisons to other nations.

Outside the general pattern, a few countries have distinctively high levels of prejudice against Gypsies: Over 60% of Slovaks would object to a Gypsy neighbor, as would over 60% of Lithuanians and Italians. Unusually for normally tolerant Anglophone nations, the Northern Irish are quite prejudiced, almost as prejudiced as the Italians.

#### The UK Has Ordinary Levels of Anti-immigrant Worker Prejudice for Its Level of Socioeconomic Development

**Figure 7:**
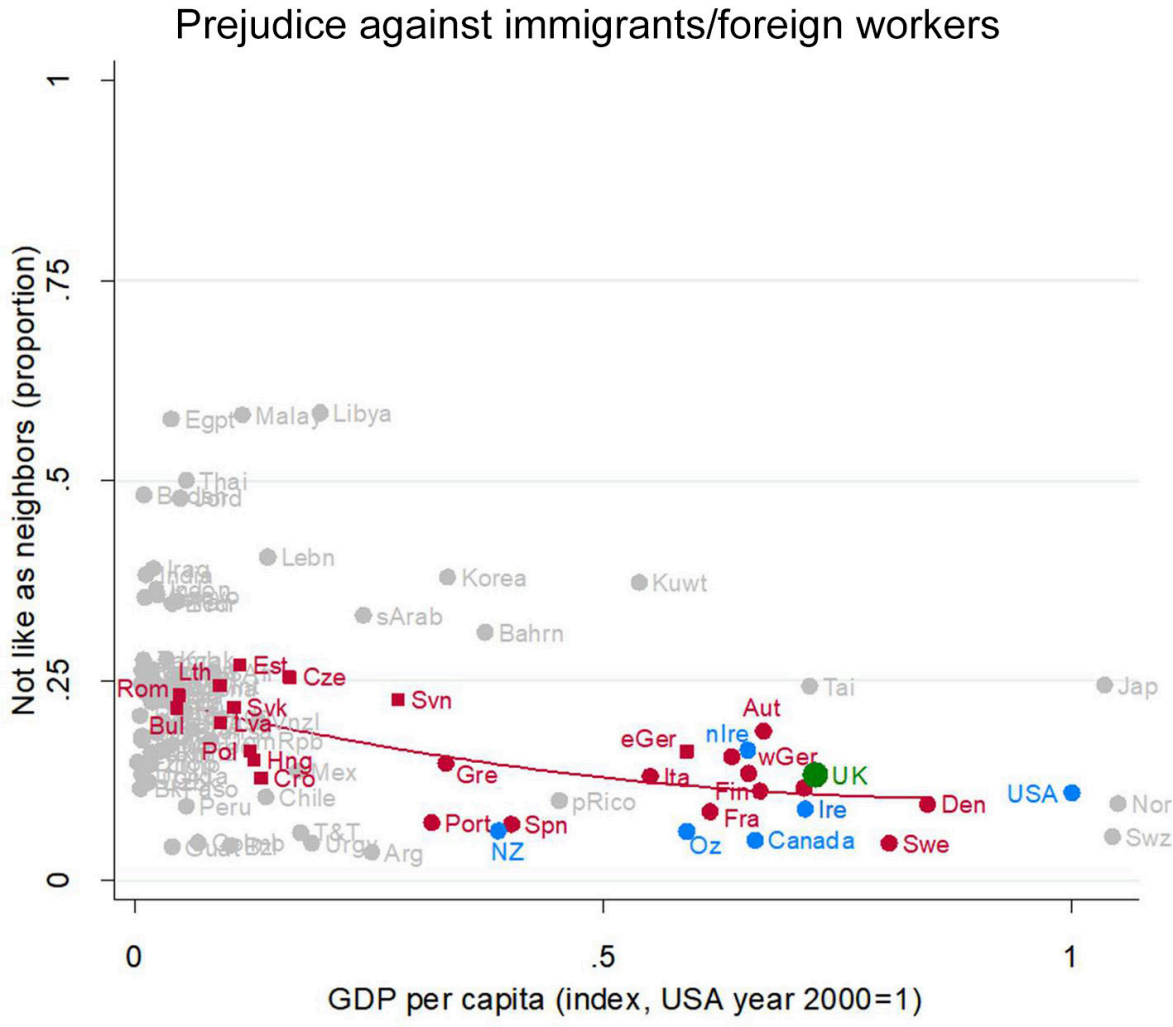
Prejudice against immigrants/foreign workers. Attitude in the UK (green), in other English-speaking nations (blue), in EU nations (red squares for ex-Communist, circles for others), and other nations (gray). Fit line just for the EU, excluding the UK. GDP per capita (index, USA year 2000 = 1). Source: World Value/European Value Studies 1981–2014 (449, 329 individuals for this analysis).

Consider first, how much prejudice against immigrant/foreign workers there is in the UK compared to peer countries in the EU. About 15% of UK residents would object to having a foreign worker as a neighbor (green dot in Figure 7, partially obscured between Austria and Sweden). This level of prejudice is exactly where we would expect an EU country at the UK level of socioeconomic development to be (the green dot sits right beside the red regression line). Thus, the UK is very similar to its peer EU countries in the prevalence of prejudice against immigrant/foreign workers. Turning to the other English-speaking countries, UK residents are fractionally more prejudiced against immigrant/foreign workers than are denizens of the other Anglophone societies, Northern Ireland excepted (compare the green dot to the blue dots).

Prejudice against immigrant/foreign workers gently declines with socioeconomic development (red regression line). Its high point is in Bulgaria, Czechia, Estonia, Lithuania, Romania, and Slovakia (with GDP per capita around 10% of US levels). There a bit over 20% object to foreign workers as neighbors. Prejudice falls slowly as GDP rises, possibly flattening out at 10–15% where GDP approaches US levels, e.g., Italy, France, Germany, Denmark. Thus, in terms of prejudice against immigrant/foreign workers, differences among EU countries, rich and poor, are rather small, and the UK is not different from the others.

But this does not mean that prejudice against immigrant/foreign workers has been tamed like an alcoholic uncle locked out of the liquor cabinet. Instead, there are clear changes over time, varying from nation to nation (a matter to which we will return).

#### One Ethno-Religious Prejudice or Many?

Thus, the UK looks like an absolutely stock standard EU country at its level of development when it comes to prejudice against each of these religious and ethnic groups. There is no sign that international labor mobility poses more of a problem to UK residents than to their EU peers.

The strong similarity of the patterns of prejudice across several of these ethnic and religious groups poses the question of whether ethno-religious prejudice is really one general attitude or many specific attitudes. There is a large literature on the matter (Semyonov et al., 2006; Cohrs and Asbrock, 2009; Scheepers and Eisinga, 2015).

To address this question, we turn to a structural equation model that will provide us with an assessment of whether it is reasonable to consider ethno-religious prejudice as a single dimension—that is a single underlying variable which all our explicit measures reflect—or whether specific prejudices are different. It will also give us a regression analysis revealing the degree to which prejudice is shaped by social location. We will restrict the prejudice variables in this analysis to those that were asked in most of the EU and Anglophone countries, so omitting results for Gypsies, Jews, and Hindus (details in Appendix B).

Ethno-religious prejudice is probably a single attitude, as shown by the measurement model results in the first column of Table 1. The confirmatory factor loadings are all substantial: 0.64–0.70. This is consistent with much previous research on the dimensionality of ethno-religious prejudice (Evans and Kelley, 1991; Agnew et al., 2000; Scheepers et al., 2002; Cohrs and Asbrock, 2009; Gorodzeisky and Semyonov, 2015). But ethno-religious prejudice is clearly distinct from prejudice on moral issues (for example, attitudes to homosexuals) and distinct from prejudice on political grounds (for example, hostility to right wing extremists). This is shown by a second confirmatory factor analysis (shaded loadings in the second column of Table 1).

**Table 1:** Alternatives to many separate prejudices, (1) a wider ethno-religious prejudice, or (2) a very broad in-group vs. out-group prejudice.

**Target of prejudice:**	**Alternative 1: Ethno-religious prejudice**	**Alternative 2: A broader in-group vs. out-group prejudice**
Immigrants/foreign workers	0.68	0.68
People of a different race	0.70	0.69
Muslims	0.65	0.66
People of a different religion	0.64	0.64
Homosexuals	–	0.30
Right wing extremists	–	0.29

*Goodness of fit measures not available because missing values are imputed*.

*Confirmatory factor loadings from structural equation analyses. Missing values imputed by maximum likelihood. World Value/European Values Studies, 1981-2014. N = 481,515*.

This model extends the range of variables to include one which is most explicitly at the heart of the labor mobility policy question: prejudice toward immigrant workers. The key point for present purposes is that prejudice against immigrant workers is a manifestation of a more general ethno-religious prejudice rather than a specific attitude about immigrant workers *per se*. This finding aligns with the ethnicity-as-cognition theory's hypothesis that ethno-religious attitudes are a coherent component of people's worldviews/schemas rather than isolated attitudes reflecting either specific experiences or historical circumstances (Brubaker et al., 2004). Imagine a 3-dimensional map with one “region” being ethno-religious prejudices: the whole region is flat (little or no prejudice) for some people, a midlevel mesa for others, and an alpine plateau for others. The whole region moves up and down together.

In almost all nations, correlations among ethno-religious prejudice items are high (Table 2, columns 4–9, below). This is especially true for the correlations between prejudice against immigrants and prejudice against other races, a pair of questions asked in almost all nations (column 4). Correlations between prejudice against immigrants and prejudice against “other religions” are equally high (column 8), although that pair of questions was not asked in quite as many nations. So too for the correlations between prejudice against immigrants and prejudice against Muslims (column 5), between prejudice against immigrants and against “other religions” (column 6), and between prejudice against other races and against Muslims. Unfortunately, we have little evidence about correlations between prejudice against Muslims and against “other religions” since that pair of questions was rarely asked (column 9).

**Table 2:** In the UK and most other developed nations mean levels of prejudice against immigrants and against other races are low (under 15% objecting to minorities as neighbors; column 3).

**Rank (mean prejudice)**	**Nation(UN code)**	**Prejudice scale (immigrants & race)**	**Correlations among ethno-religious prejudice items^a^**						**Cases (for the correlation in col._4)**
			**Immigrants & other races**	**Immigrants & Muslims**	**Immigrants & other religions**	**Other races & Muslims**	**Other races & other religions**	**Muslims & other religions**	
**(1)**	**(2)**	**(3)**	**(4)**	**(5)**	**(6)**	**(7)**	**(8)**	**(9)**	**(10)**
–	**All nations (pooled)**	**0.18**	**0.48**	**0.47**	**0.42**	**0.43**	**0.48**	**0.33**	**442,801**
1	032. Argentina	0.03	0.37	0.39	0.16	0.45	0.13	..	6,398
2	780. Trinidad & Tobago	0.03	0.24	..	0.29	..	0.21	..	2,001
3	076. Brazil	0.04	0.46	..	0.37	..	0.45	..	4,768
4	170. Colombia	0.04	0.33	..	0.30	..	0.28	..	1,512
5	554. New Zealand	0.04	0.39	..	0.22	..	0.34	..	2,996
6	858. Uruguay	0.05	0.59	..	0.40	..	0.38	..	3,000
7	320. Guatemala	0.05	0.10	0.12	..	0.13	..	..	1,000
8	036. Australia	0.05	0.47	..	0.27	..	0.32	..	6,174
9	124. Canada	0.06	0.49	0.47	0.49	0.37	0.39	0.30	7,005
10	756. Switzerland	0.06	0.50	0.34	0.50	0.35	0.56	..	5,107
11	752. Sweden	0.06	0.60	0.47	0.42	0.43	0.47	0.35	7,421
12	630. Puerto Rico	0.08	0.40	..	..	..	..	..	1,884
13	724. Spain	0.08	0.52	0.55	0.23	0.55	0.32	..	13,920
**14**	**840. United States**	**0.09**	**0.38**	**0.48**	**0.16**	**0.40**	**0.51**	..	**10,378**
15	250. France	0.09	0.50	0.60	..	0.50	..	..	5,297
16	208. Denmark	0.09	0.51	0.52	..	0.48	..	..	4,494
17	152. Chile	0.09	0.57	0.50	0.37	0.50	0.44	..	5,700
18	604. Peru	0.10	0.47	0.56	0.31	0.51	0.34	..	5,422
19	578. Norway	0.10	0.60	0.56	0.26	0.50	0.38	..	5,523
20	528. Netherlands	0.11	0.48	0.50	0.23	0.49	0.28	..	7,702
21	222. El Salvador^b^	0.11	..	..	..	..	..	..	0
22	372. Ireland	0.11	0.44	0.51	..	0.42	..	..	4,012
**23**	**826. United Kingdom**	**0.11**	**0.47**	**0.57**	**0.14**	**0.49**	**0.26**	**..**	**6,222**
24	854. Burkina Faso	0.11	0.52	..	0.44	..	0.41	..	1,534
25	620. Portugal	0.11	0.44	0.41	..	0.47	..	..	3,697
26	348. Hungary	0.12	0.40	0.44	0.23	0.41	34.00	..	4,155
27	716. Zimbabwe	0.13	0.51	0.44	0.35	0.39	0.33	..	2,502
28	**276. Germany**	**0.13**	**0.45**	**0.41**	**0.33**	**0.32**	**0.40**	**0.23**	**14,564**
29	380. Italy	0.13	0.61	0.57	0.47	0.56	0.52	..	7,784
30	246. Finland	0.14	0.50	0.49	0.50	0.38	0.55	..	5,718
31	860. Uzbekistan	0.14	0.35	..	0.35	..	0.28	..	1,500
32	909. Northern Ireland	0.14	0.53	0.64	..	0.57	..	..	2,047
33	484. Mexico	0.15	0.44	0.43	0.43	0.37	0.42	..	10,827
34	191. Croatia	0.15	0.49	0.52	0.24	0.50	0.37	..	3,564
35	158. Taiwan	0.15	0.40	..	0.29	..	0.41	..	3,245
36	300. Greece	0.15	0.51	0.49	..	0.43	..	..	2,629
37	**156. China**	**0.15**	**0.46**	**0.48**	**0.35**	**0.31**	**0.39**	**..**	**7,791**
38	070. Bosnia Herzg.	0.15	0.41	0.36	..	0.52	..	..	2,639
39	804. Ukraine	0.15	0.43	0.47	0.42	0.33	0.37	..	7,931
40	834. Tanzania	0.16	0.46	0.44	..	0.34	..	..	1,171
41	231. Ethiopia	0.16	0.53	..	0.58	..	0.52	..	1,500
42	800. Uganda	0.16	0.52	0.51	..	0.38	..	..	1,002
43	056. Belgium	0.16	0.54	0.57	..	0.49	..	..	7,350
44	643. Russia	0.16	0.40	0.47	0.33	0.43	0.43	..	12,437
45	616. Poland	0.16	0.49	0.55	0.45	0.48	0.47	..	6,639
46	586. Pakistan	0.16	0.15	..	0.05	..	0.17	..	3,200
47	040. Austria	0.17	0.52	0.51	..	0.49	..	..	4,431
48	398. Kazakhstan	0.17	0.20	..	0.22	..	0.35	..	1,500
49	112. Belarus	0.17	0.44	0.49	0.31	0.46	0.38	..	7,069
50	428. Latvia	0.18	0.33	0.41	0.42	0.39	0.20	..	4,546
51	688. Serbia	0.18	0.51	0.51	..	0.46	..	..	3,797
52	214. Dominican Rep.	0.18	0.61	..	..	..	..	..	417
53	710. South Africa	0.19	0.25	0.39	0.18	0.38	0.38	..	13,968
54	891. Serbia & Montg.	0.19	0.49	..	0.40	..	0.48	..	1,220
55	504. Morocco	0.19	0.39	..	0.29	..	0.35	..	3,646
56	862. Venezuela	0.19	0.50	..	0.58	..	0.54	..	2,400
57	646. Rwanda	0.20	0.72	..	0.72	..	0.67	..	3,034
58	914. Bosnia	0.21	0.32	..	0.34	..	0.76	..	800
59	608. Philippines	0.21	0.39	0.30	0.39	0.32	0.38	..	3,600
60	498. Moldova	0.22	0.36	0.41	0.36	0.35	0.30	..	4,508
61	788. Tunisia	0.22	0.51	..	0.48	..	0.36	..	1,205
62	392. Japan	0.22	0.62	0.52	0.40	0.41	0.38	..	4,658
63	705. Slovenia	0.22	0.60	0.67	0.50	0.66	0.55	..	6,438
64	703. Slovakia	0.22	0.37	0.45	..	0.44	..	..	5,377
65	288. Ghana	0.23	0.45	..	0.45	..	0.42	..	3,086
66	807. Macedonia	0.23	0.51	0.59	..	0.50	..	..	3,533
67	100. Bulgaria	0.23	0.47	0.47	0.45	0.45	0.47	0.51	5,465
68	203. Czech Republic	0.23	0.41	0.46	..	0.45	..	..	7,802
69	466. Mali	0.24	0.56	..	0.47	..	0.53	..	1,534
70	642. Romania	0.24	0.51	0.57	0.53	0.52	0.47	..	8,035
71	233. Estonia	0.24	0.47	0.50	0.38	0.43	0.40	..	6,007
72	566. Nigeria	0.24	0.43	0.26	0.45	0.29	0.36	..	6,778
73	440. Lithuania	0.24	0.34	0.42	..	0.35	..	..	4,518
74	031. Azerbaijan	0.25	0.48	..	0.39	..	0.34	..	3,004
75	008. Albania	0.25	0.45	0.54	0.42	0.41	0.31	..	3,346
76	417. Kyrgyzstan	0.25	0.50	0.48	0.46	0.46	0.41	..	2,543
77	268. Georgia	0.26	0.51	0.61	0.44	0.42	0.42	..	6,126
78	894. Zambia	0.28	0.26	..	0.22	..	0.34	..	1,500
79	915. Kosovo	0.29	0.47	0.31	..	0.30	..	..	1,453
80	012. Algeria	0.29	0.40	..	0.35	..	0.27	..	2,482
81	051. Armenia	0.30	0.46	−0.07	0.30	−0.18	0.37	..	4,561
82	364. Iran	0.31	0.32	..	0.38	..	0.39	..	5,196
83	048. Bahrain	0.32	0.06	..	0.22	..	0.14	..	1,200
84	414. Kuwait	0.33	0.32	..	..	..	..	..	1,303
85	792. Turkey	0.33	0.51	0.09	0.50	0.21	0.59	..	12,815
86	704. Viet Nam	0.34	0.64	0.41	0.74	0.42	0.63	..	2,495
87	218. Ecuador	0.34	0.66	..	0.62	..	0.74	..	1,202
88	360. Indonesia	0.34	0.57	..	0.52	..	0.59	..	3,003
89	368. Iraq	0.34	0.20	..	0.21	..	0.30	..	1,200
90	410. South Korea	0.35	0.62	0.37	0.45	0.27	0.44	..	5,821
91	422. Lebanon	0.37	0.29	..	0.22	..	0.34	..	1,200
92	458. Malaysia	0.37	−0.06	..	−0.04	..	0.67	..	2,500
93	682. Saudi Arabia	0.37	0.32	..	0.45	..	0.38	..	1,502
94	887. Yemen	0.37	0.45	..	0.39	..	0.38	..	1,000
95	764. Thailand	0.38	0.35	..	0.40	..	0.43	..	2,712
96	**356. India**	**0.39**	**0.35**	**0.35**	**0.39**	**0.38**	**0.46**	**..**	**10,124**
97	400. Jordan	0.40	0.39	..	0.37	..	0.43	..	3,623
98	050. Bangladesh	0.44	0.52	..	0.47	..	0.58	..	3,025
99	818. Egypt	0.46	0.38	..	..	..	..	..	3,000
100	434. Libya	0.56	0.42	..	0.43	..	0.41	..	2,131

a *All correlations are significantly different from zero at p < 0.001*.

b *El Salvador asked only one prejudice question, prejudice against other religions, so its mean is based on that*.

*In almost all nations correlations among ethno-religious prejudice items are high (columns 4–9), suggesting there are not many separate prejudices but instead a single ethno-religious prejudice. Pairwise present correlations for all available data (not every question was asked in every survey); all correlations are statistically significant at p < 0.001. Nations ranked from least prejudiced to most prejudiced. EU nations in red and English-speaking nations in blue. World Value/European Value Studies, 1981–2014. Illustrative countries are bolded*.

In all, the pattern of ethno-religious prejudice is reasonably clear in almost all nations where the questions were asked. In the few countries that are highly diverse religiously with substantial numbers of Christians, Muslims, and also other religions, differences between alternative targets of religious prejudice are probably small, but the evidence on this is sparse (Table 2, column 9). Australian evidence from a large, representative national sample suggests extremely high correlations between prejudice against various immigrants (Vietnamese. Greek, British, American) but much lower correlations for social minorities (gays, fat people, smokers; Kelley and Kelley, 2016).

We will see later that changes over time in religious prejudice, at least in the UK and the EU, may be a little different than the (mostly small and unsystematic) changes in other forms of prejudice. This raises the possibility that the immigrant/ race/ethnic components of prejudice may be somewhat different than the religious components.

By far the strongest effect on this latent ethno-religious prejudice variable is socioeconomic development as indexed by GDP per capita, as shown by the standardized coefficient of −0.17 (Table 3). The other national context characteristic we included is UK residence. Importantly for our purposes, the results demonstrate that the UK is not an outlier: UK residents hold ethno-religious prejudices no stronger than their peers in other countries at the same level of development. If anything, they are fractionally less prejudiced than are otherwise comparable people in equally developed countries, but the effect too weak to highlight. The standardized coefficient is statistically significant, but its strength is in the too-weak-to-matter zone under 0.05. Moreover, the metric effect (in green) is extremely small. A reasonable verdict would be “not substantially different, perhaps a hair less prejudiced.”

**Table 3:** Influences on ethno-religious prejudice.

	**Standardized effect**	**Significance, z**
UK (0 or 1)		
Standardized effect	−0.004	−2.1, *p* < 0.05
Metric effect	−0.008	—
GDP per capita in current dollars, year 2000	−0.17	−91.1, *p* < 0.001
Male (0 or 1)	0.03	16.0, *p* < 0.001
Age (years)	0.02	9.9, *p* < 0.001
Education (years)	−0.07	−34.9, *p* < 0.001
Family income (relative to rest of nation)	−0.02	−12.3, *p* < 0.001
Religious belief (4 item scale, alpha reliability = 0.84, scored low to high)	0.04	24.2, *p* < 0.001

*Standardized (except where indicated) effect parameters from a structural equation analysis (missing data imputed by maximum likelihood). Metric effect in green. World Value/European Values Studies, 1981–2014. N = 481,515*.

*Goodness of fit for an analogous model where missing values are not imputed (see Appendix): Root mean squared error of approximation, RMSEI = 0.02; Comparative Fit Index, CFI = 0.985*.

Turning to the individual-level characteristics, education is the most important of the personal influences on ethno-religious prejudice with a standardized coefficient of −0.07. There is some doubt that the education effect is genuinely causal, with an alternative possibility being that both educational attainment and ethnic tolerance reflect the cultural stance of the family of origin (Lancee and Sarrasin, 2015). We take no position on that issue, as education is just a control variable in this model.

Note that these results are consistent with traditional sociological theory positing that development and education undermine prejudice (Allport and Kramer, 1946; Parsons, 1964). But note also that neither of them is a really large effect: Education's effect is near the top of the conventional weak-but-worth-keeping-in-mind range (absolute value of 0.05–0.10) and GDP is in the moderately strong range (absolute value of 0.10–0.20).

The other personal characteristics' effects are significantly different from zero (this is a very large sample), but are in the nugatory, too-weak-to-matter range (significant, but absolute value of the standardized coefficient < 0.05). Otherwise put, age, relative income, and religious belief all have probably real but negligibly weak effects on ethno-religious prejudice. Gender differences also are significant and too weak to matter (Rustenbach, 2010).

#### Changes Over Time: Is Ethno-Religious Prejudice Increasing?

Thus, prejudice against immigrant/ foreign workers and against other races are thus just two of several aspects of a more general ethno-religious prejudice. But they are nonetheless worth examining on its own as they are emerging as the articulated, explicit aspect, thought by many commentators to be a divisive issue separating the UK from the rest of the EU, and important in current political discussion in many economically developed nations, including the USA. We will return to the explicitly religious aspects of prejudice in a moment.

In the UK, most other Northern European nations, and the US there are no simple linear changes over time in prejudice against immigrants or against other races between 1981 and 2014 (Table 4). Changes over time in other nations show no clear pattern: In some prejudice against immigrants and against other races increases (positive) and in others it decreases (negative). This is after adjusting for age, gender, religious belief, education, and income.

**Table 4:** There are no clear changes over time in prejudice in the UK or most other Northern European nations.

**Nation (UN code)**	**Change in prejudice, 1990 to 2020**		**EU_xUK**	**Anglo**
	**Amount**	**Significance**		
410. South Korea	−0.33	**p** < 0.001	0	0
705. Slovenia	−0.31	*p* < 0.001	1	0
100. Bulgaria	−0.28	*p* < 0.001	1	0
484. Mexico	−0.27	**p** < 0.001	0	0
703. Slovakia	− 0.19	*p* < 0.001	1	0
**156. China**	**− 0.18**	***p*** **< 0.001**	0	0
642. Romania	− 0.14	*p* < 0.001	1	0
152. Chile	−0.13	**p** < 0.001	0	0
056. Belgium	− 0.09	*p* < 0.001	1	0
348. Hungary	− 0.09	*p* < 0.001	1	0
616. Poland	− 0.09	*p* < 0.001	1	0
554. New Zealand	− 0.07	*p* < 0.001	0	1
578. Norway	−0.06	**p** < 0.001	0	0
032. Argentina	−0.05	**p** < 0.001	0	0
604. Peru	−0.04	**p** < 0.05	0	0
124. Canada	−0.02	*p* < 0.05	0	1
250. France	−0.02	ns	1	0
**276. Germany**	**− 0.01**	**ns**	1	0
246. Finland	−0.01	ns	1	0
428. Latvia	−0.01	ns	1	0
752. Sweden	0.00	ns	1	0
**826. United Kingdom**	**0.00**	**ns**	**0**	**1**
208. Denmark	0.01	ns	1	0
724. Spain	0.01	ns	1	0
036. Australia	0.02	*p* < 0.01	0	1
528. Netherlands	0.02	*p* < 0.05	1	0
**840. United States**	**0.02**	***p*** **< 0.001**	0	1
756. Switzerland	0.03	**p** < 0.01	0	0
909. Northern Ireland	0.04	ns	0	1
203. Czech Republic	0.07	*p* < 0.001	1	0
792. Turkey	0.09	**p** < 0.001	0	0
440. Lithuania	0.10	*p* < 0.001	1	0
380. Italy	0.10	*p* < 0.001	1	0
372. Ireland	0.11	*p* < 0.001	1	1
804. Ukraine	0.16	*p* < 0.001	0	0
**356. India**	**0.18**	***p*** **< 0.001**	0	0
233. Estonia	0.23	*p* < 0.001	1	0
498. Moldova	0.26	*p* < 0.001	0	0
710. South Africa	0.27	*p* < 0.001	0	0
643. Russia	0.34	*p* < 0.001	0	0
268. Georgia	0.39	*p* < 0.001	0	0
112. Belarus	0.43	*p* < 0.001	0	0

*Changes over time in other nations show no clear pattern. Estimated increase (positive) or decrease (negative) in mean prejudice against immigrants and against other races between 1990 and 2020. Predicted values from country-by-country OLS regression estimates controlling for age, gender, religious belief, education, and income; units are the same as in Table 2, column 3. Only nations with 4 or more surveys. Number of cases shown in Appendix Table A1. EU nations in red and English-speaking nations in blue. World Value/ European Value Studies, 1981–2014. Illustrative countries are bolded*.

Specifically, prejudice has dropped sharply in South Korea, Mexico, several Balkan nations in the EU, and in (authoritarian and famously racist) China. But it has increased in (democratic) India and South Africa as well as in Russia and several Eastern European nations that are outside the EU.

Rather than the simple linear patterns of change in Table 4, looking at more complex patterns of change leaves the picture equally diverse. We do this in the usual way by including a quadratic term in the model, year squared, which caters for a wide variety of patterns with a single inflection point (details in Appendix B). These results (not shown in detail but available on request) show no clear change in some nations (UK, USA); sharp declines in prejudice (China, Mexico); increasing prejudice particularly in recent years (Russia, India); and a clear U-shaped pattern with prejudice at first declining, bottoming out around the turn of the century, and then increasing in recent years (Germany, Netherlands).

**Figure 8:**
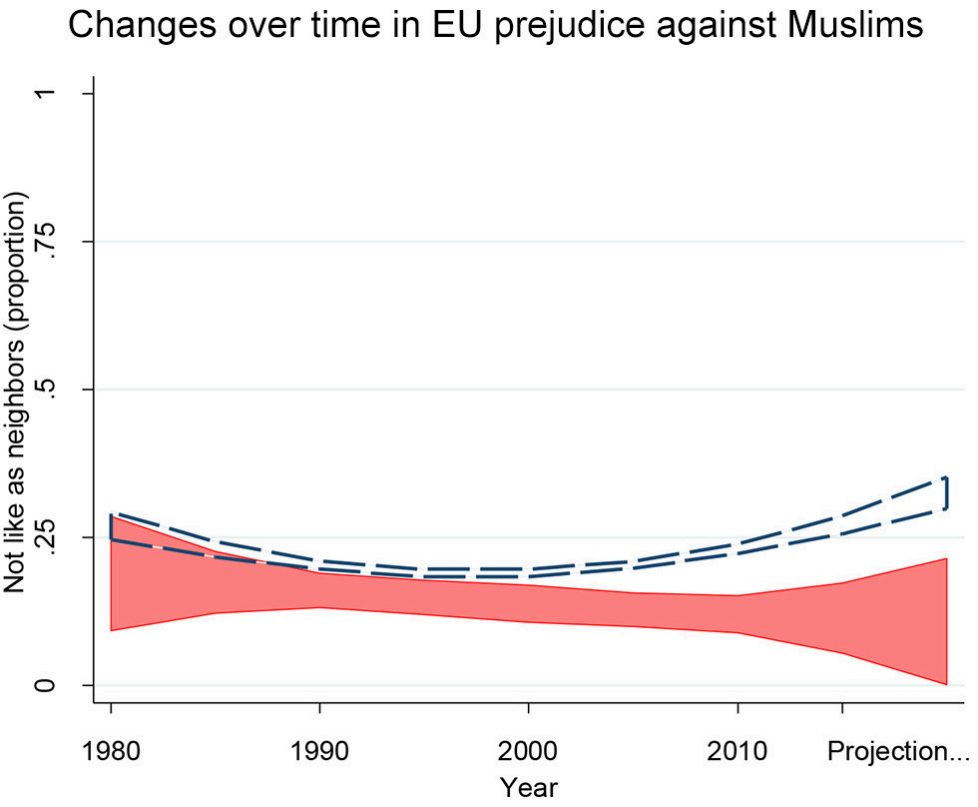
Changes over time in EU prejudice against Muslims. In the 1980s prejudice was relatively low in the UK almost as low in the rest of the EU. In the UK it then declined slowly up to the present. In the rest of the EU it also declined slowly up to the mid-90s but then has begun to increase again. Nighty five percent of confidence intervals from OLS regressions controlling age, sex, education, income, and religious belief. UK in red (*N* = 3,932) and the rest of the EU outlined in dashes (*N* = 100,325). Results after 2014 are projections from the earlier patter; details in the technical Appendix. Source: World Value/European Value Studies pooled file, 1981-2014.

Prejudice specifically against Muslims shows a somewhat different pattern^1^. In the UK it was already low in the 1980s when our data begin, fractionally lower than in other EU nations. It has declined slowly since then (see also Storm et al., 2017) and is now somewhat lower than in the rest of the EU—not a lot lower but clearly lower (Figure 8). So insofar as prejudice against Muslims contributes to hostility to the EU (which it does, slightly), Brexit is not the end of the story but merely the beginning.

For the rest of the EU outside the UK, prejudice specifically against Muslims has not, on the whole, changed much between 1980 and 2010. But there is a small, statistically significant curvilinear patterns (joint *t*-test for year of survey and its square: *F*_(2, 3, 925)_ = 4.18, *p* < 0.05). Prejudice against Muslims was not particularly great in the EU around 1980 and declined slowly up to the mid-1990s (Figure 8). But then it has likely begun to increase, again reaching its 1980 level around 2005. The natural projection would then be even greater prejudice in subsequent years. Consistent with this, Pew Research Center survey data for changes between 2015 and 2016 also show an increase in unfavorable views of Muslims both in less tolerant EU nations (Italy, Poland, Greece, Spain) and in more tolerant France and Germany. Unlike our estimates they also show an increase in the (very tolerant) UK, from 19% unfavorable to 28% unfavorable (Pew Research Center, 2016).

### Results, Part 2: Sensitivity Tests

#### Above Results Hold With Different Prejudice Measures and Other Survey Datasets

All research risks over-specificity, that the findings reflect idiosyncratic features of the particular questions we use rather than the concept we hope to measure, or idiosyncratic features of the dataset being analyzed. This makes it a priority to discover the degree to which the findings are robust across questions and datasets (Pautasso, 2010; John et al., 2012).

##### Sensitivity test #1: a different prejudice measure shows the same pattern

Consider first the WVS/EVS question on willingness to discriminate against foreigners in employment. In a different part of the questionnaire, the WVS/EVS asked: “Do you agree, disagree or neither agree nor disagree with the following statements? When jobs are scarce, employers should give priority to people of this country over immigrants.” The answer options were: “1. Agree,” “2. Neither,” and “3. Disagree.” There are 146,096 cases with valid data on this question.

**Figure 9:**
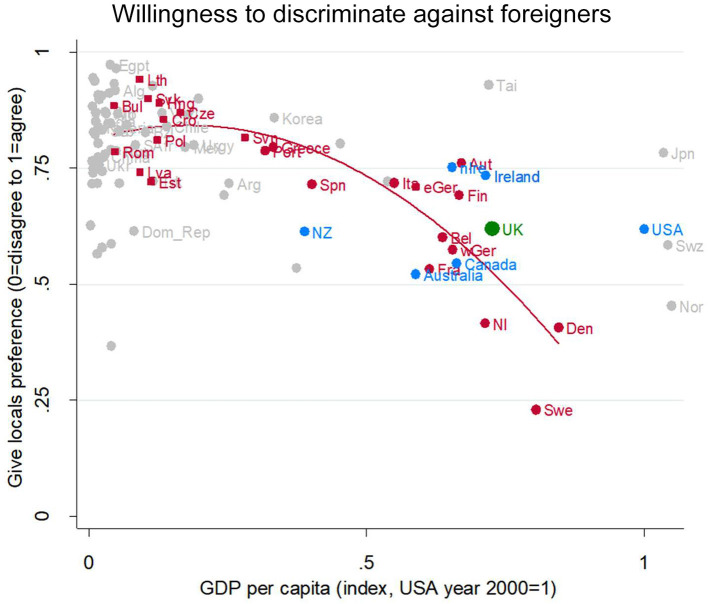
Willingness to discriminate against foreigners. Attitude in the UK (green), in other English-speaking nations (blue), in EU nation (red squares for ex-Communist, circle for others), and other nations (gray). Fit line just for the EU, excluding the UK. GDP per capita (index, USA year 2000 = 1). Source: World Value/European Value Studies 1981–2014 pooled (417, 940 individuals for this analysis).

For willingness to discriminate, like neighboring prejudice, there is a clear connection with GDP in the EU countries (Figure 9, red line and red dots): The higher the GDP, the lower the willingness to discriminate. Willingness to discriminate in the UK is not at all unusual for an EU country at its level of socioeconomic development: The UK's green dot is very near the regression line for EU countries, very similar to Belgium, and (former) West Germany (red dots). Turning to resemblance to the other Anglophone countries, the UK (green dot) is a very similar to the US and New Zealand, possibly fractionally higher than Australia and Canada and somewhat lower than Ireland (blue dots).

Thus, on this alternative measure, the UK is very similar to its peer countries in the EU. This substantiates the view that the close resemblance of the UK to other prosperous EU countries in terms of prejudice is real, rather than being an artifact of the particular neighboring questions analyzed above.

##### Sensitivity test #2: another prejudice measure in a different dataset shows the same pattern

Data for a second sensitivity test are from a well-known high-quality dataset, the European Quality of Life Survey, covering the European Union and a few nations in the process of applying. It is fully described on its website, www.eurofound.europa.eu. We analyze the 29 nations with populations over 1 million in the 2011–2012 wave; all are representative national samples. There are 66,795 respondents with the relevant information.

The question was, “How about people from other countries coming here to live and work? Which one of the following do you think the government should do?” The answer options were, “Let anyone come who wants to,” “Let people come as long as there are jobs available,” “Put strict limits on the number of foreigners who can come here to work,” “Prohibit people coming here to work.”

Analyses of differences between the UK and other nations, controlling GDP per capita and year of survey, are shown in Table 5, and compared to our previous analysis.

**Table 5:** Sensitivity tests: (#1) Different measure of discrimination, same data set; (#2) yet another measure of discrimination and an entirely different dataset.

	**Original analysis**	**Test #1**	**Test #2**
	Question: Prejudice against Immigrants/ foreign workers	Question: Willingness to discriminate against foreigners	Question: Favor restricting immigration
	Data: World Value Study/ European Values Study. 1981–2014. EU and UK.	Data: World Value Study/ European Values Study. 1981–2014. EU and UK.	Data: European Quality of Life Survey, 2011. EU and UK.
UK (0 or 1)	0.01	0.03	0.11
GDP per capita	−0.11^***^	−0.31^***^	−0.06^*^
R-squared	0.01	0.10	0.01
Rho (country variance)	0.02	0.06	0.08
Number of countries	26	26	29
Number of cases	160,695	146,096	66,795

* *p < 0.05*,

^**^p < 0.01,

*** *p < 0.001*.

*Metric regression coefficients from multi-level variance components models estimated by GLS. UK and European Union nations only*.

The UK is not distinctive in any of these alternative analyses (row 1, highlighted). All the UK dummy variable effects are non-significant at *p* < 0.05. The results are from multilevel regressions, specifically variance-components models with individual-level fixed effects and random intercepts, estimated by GLS.

These replications also suggest that the pattern of prejudice declining with socioeconomic development may be rather general—it is statistically significant in both replications—but of varying magnitude. In Sensitivity Test #1, the decline is much larger than in our main model, but in Sensitivity Test #2 it is still significant, but smaller in magnitude.

The crucial point for present purposes is that the UK closely resembles peer countries in the EU using different questions and different datasets. That inspires confidence that the resemblance is real.

Furthermore, we included the percent of immigrants as a country-level variable in the model. In a multilevel analysis (estimated, like the previous models in STATA's xtreg via GLS with fixed effects and random intercepts) of the 48 countries with available data, with 64,865 individual cases. The effect was not statistically significant (*Z* = −0.19; *p* = 0.847). Including this higher-level variable did not change the GDP effects. This is not necessarily strong evidence against the Contact Hypothesis: (1) contact may reduce prejudice as much prior research suggests (Hewstone and Swart, 2011), but status threat effects (Davidov and Semyonov, 2017) may also be present at the same time and they may cancel each other out; (2) the national percent of immigrants is, at best, a weak indicator of contact, because residential and social segregation may severely restrict social interaction.

## Discussion

### Summary: Public Attitudes Toward Migrants Symptomatize a Syndrome of Attitudes Toward Minority Ethno-Religious Groups; Socioeconomic Development Is a Major Determinant of Ethno-Religious Prejudice; The UK Is Not Exceptional; Prejudice on the Rise in Some Countries (India, Russia), Stable in Some (UK, Germany), and Declining in Others (China, Mexico)

All these results point to the UK as being a very typical prosperous EU country when it comes to ethno-religious prejudice generally, and specifically to prejudice against immigrant/foreign workers. A well-established indicator of prejudice—the desire to shun a certain type of person as a neighbor—shows that the British levels of prejudice against immigrant/foreign workers and people “of a different race,” Hindus, Jews, Gypsies, and especially Muslims are all relatively low. Moreover, British prejudice levels are just where they would be expected to be based on the general pattern of prejudice and socioeconomic development within the EU. They are also generally close to the other Anglophone countries. We find a general pattern of high prejudice and desire to discriminate in the less advanced countries with a strong decline in these emotions with socioeconomic development (to which the UK conforms) as predicted by the hierarchy of needs/post-materialist approach (Maslow, 1943; Inglehart, 1981; Inglehart and Welzel, 2010).

But it cannot be taken for granted that these largely benign attitudes toward foreign workers will persist or even improve. In some places, net of development, prejudice appears to be decreasing, in others holding steady, and in others increasing. There is no obvious pattern.

Another issue is the explicit desire to discriminate against foreign workers in hiring and employment. If this were an important impediment to collaboration between the UK and the EU, we would expect that the desire to discriminate is stronger in the UK than elsewhere. But in fact, there is nothing exceptional about the UK in this (Sensitivity Test #1). Nor does the UK show any unusual preference for restricting immigration (Sensitivity Test #2).

### Working Hypotheses

The results suggest several working hypotheses to be addressed in future research:

H1: Prejudices against all religious and ethnic outgroups all reflect a single latent ethno-religious prejudice variable.
H2: Ethno-religious prejudice is distinct from prejudices against other outgroups.
H3: Socioeconomic development has a positive, albeit not hugely strong effect increasing ethno-religious tolerance.
H4: The UK is not distinctive in ethno-religious prejudice, net of socioeconomic development and social composition (individual characteristics).

### Discussion: Implications of Attitudes Toward Immigrants as a Symptom of a Broader Ethno-Religious Prejudice Dimension

This similarity to the general EU pattern suggests that public opinion in the UK about foreign workers is no more of an obstacle to a common market than is true for other European countries. Thus, if it was one cause of Brexit (as is likely), it is a cause that could apply equally to many other EU nations in future years.

The relatively low levels of prejudice in most of the EU for this whole period are not grounds for complacency. An in-depth study in the Netherlands suggests that exposure to immigrants may have a u-shaped (concave up quadratic; down, then up) effect on prejudice (Havekes et al., 2011), although, in general, there is strong support for the “contact hypothesis” (Hewstone and Swart, 2011). Perhaps the presence of immigrants has strong ambivalent effects, with increasing availability of immigrants as interaction partners reducing prejudice and, at the same time, status threat increasing prejudice (Davidov and Semyonov, 2017): The balance between the two effects may be unstable and could tilt suddenly. If so, rapid changes may occur throughout the EU in the future—sharp drops among the more prejudiced countries and sharp rises among those who have passed the “sweet spot.” If public response to immigration was one of the troubles leading the UK to leave the EU, there is fertile ground in public opinion for similar troubles in the years to come in Germany, the Netherlands and perhaps elsewhere.

However, ethno-religious prejudice does not translate directly into party politics not only because it is only one issue among many, but also because all the parties seem to have slightly shifted in an anti-immigrant direction which seems to have preserved adherence to the major parties among mildly prejudiced people (Wagner and Meyer, 2016).

The finding that ethno-religious prejudice is really one attitude with many symptoms suggests important possibilities for beneficial and harmful effects on social cohesion and harmony in the future. In particular, in a kind of extended version of the Contact Hypothesis (Allport, 1979 [1954]; Pettigrew and Tropp, 2006; Hewstone and Swart, 2011) positive contact with a member of one minority group is likely to erode prejudice against members of all ethno-religious minority groups Moreover, this finding, in conjunction with knowledge of the availability heuristic (Tversky and Kahneman, 1973) suggests that terrorist attacks against the majority population by members of any such group would be likely to stimulate prejudice against all potential outgroups in the ethno-religious domain. Moreover, the result that anti-immigrant feeling is really not a separate thing, but rather a symptom of a latent ethno-religious prejudice against a wide variety of such groups reinforces the emerging understanding that issues of framing, national identity, values for cultural distinctiveness, and nonlinear cultural trends play an important role influencing prejudice levels now and possibly an even more important role shaping future trends in prejudice in the advanced societies (Davidov and Semyonov, 2017).

Of course, even though simple issues of status threat seem to be less important than originally thought (Kuntz et al., 2017 and our final sensitivity test), stratification and hierarchy still matter to prejudice (Jasso, 2011, 2014), but perhaps somewhat differently than atomistic economic self-interest theories suggest. The key issue may be the degree to which status as a good and valuable person requires adherence to specific cultural practices: If these are required, that puts such status within the reach of locals even with few economic and cognitive resources; if they are not required—as potentially evidenced by elite tolerance of ethno-religious outgroups or economic success of these outgroups—access to status as a good and valuable person is potentially harder to achieve for locals with few economic and cognitive resources. In other words, the potential link between cultural “tightness” or closure and prejudice may be quite different for members of the dominant group according to their social class/stratification position (Davidov and Semyonov, 2017).

Further grounds for concern are that terrorist attacks of recent years, in addition to their immediate and direct harm, may impair generalized social trust. That matters to the future of European cooperation because social trust influences prejudice (Rustenbach, 2010). Also, to the extent that such incidents are associated in the public mind with any ethnic and or religious group and stimulate prejudice against that group, this could ramify into increased ethno-religious prejudice across the board. On the other hand, ongoing socioeconomic development and advancing educational attainment are likely to reduce prejudice against all potential outgroups in the ethno-religious domain (to the extent that the observed relationships are causal, which is plausible but beyond the scope of the present paper to establish). We have discussed socioeconomic development as indexed by GDP in terms of a Maslowian interpretation (see also Inglehart and Welzel, 2005), but it is also possible that socioeconomic development may enhance tolerance of ethno-religious outgroups by attracting migrants to the country and thereby increasing positive contact and reducing prejudice against all ethno-religious minorities.

All in all, prejudice and willingness to discriminate against foreign workers are relatively low. They are rising in some European nations—despite a countervailing trend of tolerance increasing with GDP—but not in others. In this, the UK is unexceptional, except perhaps that prejudice against Muslims may be a little lower than in peer countries in the EU. This strongly suggests that Brexit did not come about because the UK's population is distinctively prejudiced and that similar issues may well-arise in other EU nations in future years. The links to right-wing populist politics will continue to demand researchers' attention.

## Data Availability

The datasets analyzed in this study can be found at www.gesis.org/en/services/data-analysis/international-survey-programs/european-values-study/, and https://www.eurofound.europa.eu/surveys/european-quality-of-life-surveys.

## Author Contributions

ME and JK wrote and argued over every part of this paper. It is part of a collaboration on analysis of immigrant issues extending over 35 years. Their contributions are indistinguishable.

### Conflict of Interest Statement

The authors declare that the research was conducted in the absence of any commercial or financial relationships that could be construed as a potential conflict of interest.

## Footnotes

^1^These results are from individual-level OLS estimates predicting prejudice from year of survey and year squared, controlling for gender, age, education, and religious belief. For the UK there are too few time periods to control for GDP per capita. For comparability GDP is also omitted for the EU nations; multilevel estimates including it are almost identical save perhaps for a fractionally sharper upturn in prejudice in recent years. Further details are available on request.

## References

[B1] Allport G. W. (1979 [1954]). [1954]. The Nature of Prejudice: 25th Anniversary Edition. New York, NY: Basic Books.

[B2] Agnew C. R. Thompson V. D. Gaines S. O. Jr. (2000). Incorporating proximal and distal influences on prejudice. Personal. Soc. Psychol. Bull. 26, 403–418. 10.1177/0146167200266001

[B3] Allport G. W. Kramer B. M. (1946). Some roots of prejudice. J. Psychol. 22, 9–39. 10.1080/00223980.1946.9917293 20992067

[B4] Bergh R. Akrami N. Sidanius J. Sibley C. G. (2016). Is group membership necessary for understanding generalized prejudice? A re-evaluation of why prejudices are interrelated. J. Personal. Soc. Psychol. 111, 367–395. 10.1037/pspi0000064 27560611

[B5] Blalock H. M. (1967). Towards A Theory of Minority Group Relations. New York, NY: John Wiley and Sons.

[B6] Bogardus E. S. (1933). A social distance scale. Sociol. Soc. Res. 17, 265–271.

[B7] Bollen K. A. (1989). Structural Equations with Latent Variables. New York, NY: Wiley.

[B8] Breznau N. (2016). Secondary observer effects: idiosyncratic errors in small-N secondary data analysis. Int. J. Soc. Res. Methodol. 19, 301–318. 10.1080/13645579.2014.1001221

[B9] Breznau N. Eger M. A. (2016). Immigrant presence, group boundaries, and support for the welfare state in western european societies. Acta Sociol. 59, 195–214. 10.1177/0001699316645168

[B10] Breznau N. Hommerich C. (2018). No generalizable effect of income inequality on public support for redistribution among rich democracies, 1980-2010. Int. J. Soc. Wlfare. 10.31235/osf.io/dhekw 31130195

[B11] Brubaker R. Loveman M. Stamatov P. (2004). Ethnicity as Cognition. Theory Soc. 33:31–64. 10.1023/B:RYSO.0000021405.18890.63

[B12] Bulmer M. Solomos J. (2010). Muslim minorities in western europe. Ethnic Racial Stud. 33, 373–375. 10.1080/01419871003589758

[B13] Castles F. G. (2010). The English-speaking countries, in The Oxford Handbook of the Welfare State, eds Castles F. G. Leibfried S. Lewis J. Obinger H. Pierson C. (Oxford: Oxford Handbooks). 10.1093/oxfordhb/9780199579396.003.0043

[B14] Cohrs J. C. Asbrock F. (2009). Right-wing authoritarianism, social dominance orientation and prejudice against threatening and competitive ethnic groups. Eur. J. Soc. Psychol. 39, 270–289. 10.1002/ejsp.545

[B15] Davidov E. Semyonov M. (2017). Attitudes toward immigrants in European societies. Int. J. Comp. Sociol. 58 359–366. 10.1177/0020715217732183

[B16] Díez-Medrano J. (2011). Building a Five Wave WVS-EVS Aggregate File from Existing Official Files Available on the Web. Vol. ASEP/JDS, Madrid: WVS Data Archive.

[B17] DiMaggio P. (1997). Culture and cognition. Ann. Rev. Sociol. 23, 263–287. 10.1146/annurev.soc.23.1.263

[B18] Esping-Andersen G. Nedoluzhko L. (2017). Inequality equilibria and individual well-being. Soc. Sci. Res. 62, 24–28. 10.1016/j.ssresearch.2016.12.010 28126102

[B19] Evans M. D. R. Kelley J. (1991). Prejudice, discrimination, and the labor market. Am. J. Sociol. 97, 721–759. 10.1086/229818

[B20] Evans M. D. R. Kelley J. (2017). Communism, capitalism, and images of class: effects of reference groups, reality, and regime in 43 Nations and 110,000 individuals, 1987-2009. Cross Cultural Res. 51, 315–359. 10.1177/1069397116677963

[B21] Evans M. D. R. Kelley J. (2018). Strong welfare states do not intensify public support for income redistribution, but even reduce it among the prosperous: a multilevel analysis of public opinion in 30 countries. Societies 8, 1–53. 10.3390/soc8040105

[B22] EVS (2015). European Values Study Longitudinal Data File 1981-2008 (Evs 1981-2008). Za4804 Data File Version 3.0.0, Cologne: GESIS Data Archive.

[B23] Fernandez J. Jaime-Castillo A. M. (2018). The institutional foundation of social class differences in pro-redistribution attitudes: a cross-national analysis, 1985–2010. Social Forces 96, 1009–1038. 10.1093/sf/sox068

[B24] Gjeddebaek N. F. (1968). Grouped observations, in Record number is 50 in International Encyclopedia of the Social Sciences, ed Sills D. S. (New York, NY: Macmillan Co. and Free Press). Available online at: http://www.cabdirect.org/abstracts/19691803250.html;jsessionid=0EDFCEBE343F124C35B918C58523664C

[B25] Gorodzeisky A. Semyonov M. (2015). Not only competitive threat but also racial prejudice: sources of anti-immigrant attitudes in european societies. Int. J. Public Opin. Res. 28, 331–354. 10.1093/ijpor/edv024

[B26] Guimond S. Dambrun M. Michinov N. Duarte S. (2003). Does social dominance generate prejudice? Integrating individual and contextual determinants of intergroup cognitions. J. Personal. Soc. Psychol. 84, 697–721. 10.1037/0022-3514.84.4.697 12703644

[B27] Haitovsky Y. (2001). Grouped data, in International Encyclopedia of the Social and Behavioral Sciences, ed. Baltes P. B. (New York, NY: Elsevier), p. 14995–5002. Available online at: http://www.sciencedirect.com/science/referenceworks/9780080430768

[B28] Havekes E. Uunk W. Gijsberts M. (2011). Explaining ethnic outgroup feelings from a multigroup perspective: similarity or contact opportunity? Soc. Sci. Res. 40, 1564–1578. 10.1016/j.ssresearch.2011.06.005

[B29] Heath A. F. Rothon C. Kilpi E. (2008). The second generation in western europe: education, unemployment, and occupational attainment. Ann. Rev. Sociol. 34, 211–235. 10.1146/annurev.soc.34.040507.134728

[B30] Heitjan D. (1989). Inference from grouped continuous data. Stat. Sci. 4, 164–179. 10.1214/ss/1177012601

[B31] Hewstone M. Swart H. (2011). Fifty-odd years of inter-group contact. Br. J. Soc. Psychol. 50, 374–386. 10.1111/j.2044-8309.2011.02047.x 21884537

[B32] Ignacz S. (2018). The remains of the socialist legacy: the influence of socialist socialization on attitudes toward income inequality. Societies 8:62. 10.3390/soc8030062

[B33] Inglehart R. (1981). Post-materialism in an environment of insecurity. Am. Polit. Sci. Rev. 75, 880–900.

[B34] Inglehart R. (1990). Culture Shift in Advanced Industrial Society. Princeton, NJ: Princeton University Press.

[B35] Inglehart R. (1997). Modernization and Postmodernization. Princeton, NJ: Princeton University Press.

[B36] Inglehart R. (2008). Mapping global values. Compar. Sociol. 5, 115–136. 10.1163/156913306778667401

[B37] Inglehart R. Welzel C. (2005). Modernization, Cultural Change, and Democracy. Cambridge: Cambridge University Press.

[B38] Inglehart R. Welzel C. (2010). The WVS Cultural Map of the World. Available online at: http://www.worldvaluessurvey.org/WVSContents.jsp?CMSID=Findings

[B39] Jasso G. (2011). Migration and stratification. Soc. Sci. Res. 40, 1292–1336. 10.1016/j.ssresearch.2011.03.007 26321771 PMC4552341

[B40] Jasso G. (2014). The social psychology of immigration and inequality, in Handbook of the Social Psychology of Inequality, eds. McLeod J. Lawler E. Schwalbe M. (Dordrecht: Springer), 575–605.

[B41] John L. K. Loewenstein G. Prelec D. (2012). Measuring the prevalence of questionable research practices with incentives for truth telling. Psychol. Sci. 23, 524–532. 10.1177/0956797611430953 22508865

[B42] Kelley J. de Graaf N. D. (1997). National context, parental socialization, and religious belief: results from 15 nations. Am. Sociol. Rev. 62, 639–659. 10.2307/2657431

[B43] Kelley J. Evans M. D. R. (1995). Class and class conflict in six western nations. Am. Sociol. Rev. 60, 157–178. 10.2307/2096382

[B44] Kelley J. Evans M. D. R. (2015). Prejudice, exclusion and economic disadvantage: a theory. Sociol. Theory 33, 201–233. 10.1177/0735275115603091

[B45] Kelley J. Evans M. D. R. (2017). The new income inequality and well-being paradigm. Soc. Sci. Res. 62, 39–74. 10.1016/j.ssresearch.2016.12.007 28126114

[B46] Kelley J. Evans M. D. R. Lowman J. Lykes V. (2017). Group-mean-centering independent variables in multilevel models is dangerous. Qual. Quant. 51, 261–228. 10.1007/s11135-015-0304-z

[B47] Kelley S. M. C. Kelley J. (2016). Attitudes towards ethnic minorities, homosexuals, smokers and the overweight: australian evidence, in Annual Meeting of the American Sociological Association (Seattle, WA). Available online at: http://citation.allacademic.com/meta/p1122905_index.html

[B48] Kunovich R. M. (2004). Social structural position and prejudice: an exploration of cross-national differences in regression slopes. Soc. Sci. Res. 33, 20–44. 10.1016/S0049-089X(03)00037-1

[B49] Kuntz A. Davidov E. Semyonov M. (2017). The dynamic relations between economic conditions and anti-immigrant sentiment. Int. J. Compar. Sociol. 58, 392–415. 10.1177/0020715217690434

[B50] Lancee B. Sarrasin O. (2015). Educated preferences or selection effects? A longitudinal analysis of the impact of educational attainment on attitudes towards immigrants. Eur. Sociol. Rev. 31, 490–501. 10.1093/esr/jcv008

[B51] Lemmer G. Wagner U. (2015). Can we really reduce ethnic prejudice outside the lab? A meta-analysis of direct and indirect contact interventions. Eur. J. Soc. Psychol. 45, 152–168. 10.1002/ejsp.2079

[B52] Lenski G. (1961). The Religious Factor. Garden City, NJ: Doubleday.

[B53] Maslow A. H. (1943). A theory of human motivation. Psychol. Rev. 50, 370–396. 10.1037/h0054346

[B54] McLaren L. M. (2003). Anti-immigrant prejudice in europe: contact, threat perception, and preferences for the exclusion of migrants. Soc. Forces 81, 909–936. 10.1353/sof.2003.0038

[B55] Miranda D. Castillo J. C. Cumsille P. (2018). The political socialization of attitudes toward equal rights from a comparative perspective, in Teaching Tolerance in a Globalized World, eds. Sandoval-Hernández A. Isac M. Miranda D. (Cham: Springer).

[B56] Neckerman K. M. Kirschenman J. (1991). Hiring strategies, racial bias, and inner-city workers. Soc. Prob. 38, 433–447. 10.2307/800563

[B57] Ng W. Diener E. (2018). Affluence and subjective well-being: does income inequality moderate their associations? Appl. Res. Qual. Life 1–26. 10.1007/s11482-017-9585-9

[B58] Park R. E. (1924). The concept of social distance as applied to the study of racial attitudes and racial relations. J. Appl. Sociol. 8, 339–344.

[B59] Park R. E. Burgess E. W. (1921). Introduction to the Science of Sociology. Chicago: University of Chicago Press.

[B60] Parsons T. (1964). Evolutionary universals in society. Am. Sociol. Rev. 29, 339–340. 10.2307/2091479

[B61] Pautasso M. (2010). Worsening file-drawer problem in the abstracts of natural, medical and social science databases. Scientometrics 85, 193–202. 10.1007/s11192-010-0233-5

[B62] Pettigrew T. F. Tropp L. R. (2006). A meta-analytic test of intergroup contact theory. J. Personal. Soc. Psychol. 90, 751–783. 10.1037/0022-3514.90.5.751 16737372

[B63] Pew Research Center (2016). Europeans Fear Wave of Refugees Will Mean More Terrorism, Fewer Jobs. Available online at: http://www.pewglobal.org/2016/07/11/europeans-fear-wave-of-refugees-will-mean-more-terrorism-fewer-jobs/

[B64] Quillian L. (1995). Prejudice as a response to perceived group threat: population composition and anti-immigrant and racial prejudice in europe. Am. Sociol. Rev. 60, 586–611. 10.2307/2096296

[B65] Roex K. L. A. Huijts T. Sieben I. (2018). Attitudes towards income inequality: ‘Winners’ Versus ‘Losers’ of the perceived meritocracy. Acta Sociol. 62, 47–63. 10.1177/0001699317748340

[B66] Ruist J. (2016). How the macroeconomic context impacts on attitudes to immigration. Soc. Sci. Res. 60, 125–134. 10.1016/j.ssresearch.2016.04.018 27712673

[B67] Rustenbach E. (2010). Sources of negative attitudes toward immigrants in europe: a multi-level analysis. Int. Migr. Rev. 44, 53–77. 10.1111/j.1747-7379.2009.00798.x

[B68] Scheepers P. Gijsberts M. Coenders M. (2002). Ethnic exclusionism in european countries - public opposition to civil rights for legal migrants as a response to perceived ethnic threat. Eur. Sociol. Rev. 18, 17–34. 10.1093/esr/18.1.17

[B69] Scheepers P. L. H. Eisinga R. N. (2015). Religiosity and Prejudice against Minorities, in International Encyclopedia of the Social and Behavioral Sciences (2nd Ed.), ed. Wright J. D. (Amsterdan: Elsevier), 381–83.

[B70] Semyonov M. Raijman R. Gorodzeisky A. (2006). The rise of anti-foreigner sentiment in european societies, 1988-2000. Am. Sociol. Rev. 71, 426–449. 10.1177/000312240607100304

[B71] Sperber D. (1985). Anthropology and psychology: towards an epidemiology of representations. Man 20, 73–89. 10.2307/2802222

[B72] Stangor C. Sullivan L. A. Ford T. E. (1991). Affective and cognitive determinants of prejudice. Soc. Cogn. 9, 359–380. 10.1521/soco.1991.9.4.359

[B73] Storm I. Sobolewska M. Ford R. (2017). Is ethnic prejudice declining in Britain? Change in social distance attitudes among ethnic majority and minority Britons. Br. J. Sociol. 68, 410–434. 10.1111/1468-4446.12250 28462536

[B74] Stouffer S. A. (1955). Communism, Conformity, and Civil Liberties. New York, NY: Doubleday.

[B75] Treiman D. J. (2009). Scale construction, in Quantitative Data Analysis, (San Francisco: Jossey-Bass), 241–62.

[B76] Tversky A. Kahneman D. (1973). Availability: a heuristic for judging frequency and probability. Cogn. Psychol. 5, 207–232. 10.1016/0010-0285(73)90033-9

[B77] Wagner M. Meyer T. M. (2016). The radical right as niche parties? The ideological landscape of party systems in western europe, 1980–2014. Polit. Stud. 65, 84–107. 10.1177/0032321716639065

[B78] Wark C. Galliher J. F. (2007). Emory bogardus and the origins of the social distance scale. Am. Sociol. 38, 383–395. 10.1007/s12108-007-9023-9

[B79] WVS (2014). World Values Survey Wave 5 2005-2008 Official Aggregate, V.20140429. Aggregate File Producer: Asep/Jds. Madrid, SpainWorld Values Survey Association, Available online at: www.worldvaluessurvey.org

[B80] WVS (2015). World value survey 1981-2014 official aggregate, in V.20150418: Wvs_Longitudinal_1981-2014_Stata_Dta_V_2015_04_18, ed. Aggregate M. S. File Producer: JDSystems. (Madrid: World Values Survey Association), Available online at: www.worldvaluessurvey.org

[B81] Zick A. Pettigrew T. F. Wagner U. (2008a). Ethnic prejudice and discrimination in europe. J. Soc. Issues 64, 233–251. 10.1111/j.1540-4560.2008.00559.x

[B82] Zick A. Wolf C. Küpper B. Davidov E. Schmidt P. Heitmeyer W. (2008b). The syndrome of group-focused enmity: the interrelation of prejudices tested with multiple cross-sectional and panel data. J. Soc. Issues 64, 363–383. 10.1111/j.1540-4560.2008.00566.x

