# Supplementary material for: Prejudice Against Immigrants Symptomizes a Larger Syndrome, Is Strongly Diminished by Socioeconomic Development, and the UK Is Not an Outlier: Insights From the WVS, EVS, and EQLS Surveys
Source: Front Sociol. 2019 Feb 26;4:12. doi: 10.3389/fsoc.2019.00012 (PMC8022649; doi:10.3389/fsoc.2019.00012)
Supplement: Supplementary file 1 [file Data_Sheet_1.docx]

**Appendix A: Details for Figures 1 through 8**

**Appendix B: Technical details**

Variable names used in this appendix

**Variable**

**pImmig Neighbours: Immigrants/foreign workers**

**pRace Neighbours: People of a different race**

**pMuslim Neighbours: Muslims**

**pOthRelg Neighbours: People of a different religion**

**uk UK**

**gnp2000 GDP per capita, year 2000, index USA=1, current dollars.**

**male Sex**

**age Age**

**ed Education, in years**

**inc income (deciles, rescaled 0 to 1} (EVS wave 4 not fully comparable)**

**belief8 Religosity, importance of religion (4 item scale; alpha= .84)**

**pEthRel9 Ethnic & racial prejudice (scale, 2 items: pImmig & pRace)**

**year330 Year of survey centered on 1900, divided by 30**

**countryUN Countery code (United Nations)**

Structural equation model (Table 1 & with slight modifications Table 3): Stata 15 code

sem ///

( pImmig pRace pMuslim pOthRelg <- ETH ) /// measurement: factor loadings

(ETH <- uk gnp2000 male age ed inc belief8) /// structural equation

, standardized nocapslatent latent(ETH) method(mlmv)

Time changes, linear (Table 4): Stata 15 code

bysort countryUN: reg pEthRel9 years30 male age ed inc belief8 if has4waves==1 & svyHasBothItems==1 , b

The non-linear patterns discussed in the text following Table 4 are from a series of country-by-country analyses adding a quadratic term for year 30. For example: (Stata 15 code)

reg pEthRel9 year00 year00Sq male age ed inc belief8 if countryUN==156, b

test year00 year00Sq

eval6 linear year00 "80 100 120" if countryUN==156

Changes in prejudice in the UK and EU (Figure 9): Stata 15 code

reg pMuslim year00 year00Sq male age ed belief8 if UK ==1 ,b

test year00 year00Sq

eval6 linear year00 "80 85 90 95 100 105 110 115 120" if UK ==1

reg pMuslim year00 year00Sq male age ed belief8 if EU_xUK ==1 ,b

test year00 year00Sq

eval6 linear year00 "80 85 90 95 100 105 110 115 120" if UK ==1
